# Inter- and intraspecific morphometric divergences in a Seychelles endemic gecko genus

**DOI:** 10.1186/s12862-026-02517-9

**Published:** 2026-04-21

**Authors:** Markus A. Roesch, Nancy Bunbury, D. James Harris, Sara Rocha, Christopher N. Kaiser-Bunbury, David J. Gower, Greg Berke, Christina Marques, Gérard Rocamora, Anna Zora, Karolin Engelkes

**Affiliations:** 1 CIBIO Research Centre in Biodiversity and Genetic Resources, InBIO Associate Laboratory, University of Porto, Vairão, 4485-661 Portugal; 2 Department of Biology, Faculty of Sciences, University of Porto, 4169-007 Porto, Portugal; 3 BIOPOLIS Program in Genomics, Biodiversity and Land Planning, Campus de Vairão, 4485-661 Vairão, Portugal; 4 Seychelles Islands Foundation, La Ciotat Building, Mont Fleuri, P.O. Box 853, Victoria, Mahé, Seychelles; 5 Centre for Ecology and Conservation, Faculty of Environment, Science and Economy, University of Exeter, Penryn Campus, Cornwall, TR10 9FE, UK; 6 Institute of Marine Research, CSIC, Eduardo Cabello 6, 36208 Vigo, Galicia, Spain; 7 Herpetology, Natural History Museum, Cromwell Road, London, SW7 5BD UK; 8 Island Biodiversity and Conservation centre, University of Seychelles, P.O. Box 1348, Anse Royale, Mahé, Seychelles; 9 Island Conservation Society, P.O. Box 775, Pointe Larue, Mahé, Seychelles; 10 Cousine Island Company Limited, Cousine Island, P.O. Box 6048, Providence, Mahé, Seychelles; 11 Frégate Island Foundation, Angelfish Marina, Roche Caiman, Mahé, Seychelles; 12 Centre for Taxonomy and Morphology, Leibniz Institute for the Analysis of Biodiversity Change, 20146 Hamburg, Germany

**Keywords:** *Ailuronyx*, Anatomy, Cranium, Cryptic species, Mandible, Micro-computed tomography, Gekkonidae, Geometric morphometrics, Multivariate morphometrics, Western Indian Ocean

## Abstract

Accurate species delimitation is essential for understanding biodiversity patterns and guiding conservation, yet cryptic species with minimal morphological variation often obscure the assessment of true species diversity, making the identification of reliable diagnostic characters critical. Island radiations exemplify this challenge by concentrating endemic diversity in restricted areas, which accentuates both the urgency of conservation and the need for accurate taxonomy, while often presenting species divergences masked by subtle morphological differences. Here, we investigated patterns of morphological differentiation in *Ailuronyx* geckos, a genus endemic to the Seychelles archipelago, comprising deeply divergent lineages and unresolved, possibly cryptic species boundaries. We applied 3D and 2D morphometric analyses across the range (16 islands) of the three recognised species: the bronze-eyed gecko *A. seychellensi*s, dwarf bronze gecko *A. tachyscopaeus*, and giant bronze gecko *A. trachygaster*. 3D geometric morphometric analyses of cranial and mandibular landmark data from 42 geckos revealed strong species-level differences. *Ailuronyx trachygaster* exhibits extreme skull sculpturing with rugose textures on up to ten cranial and three mandibular bones, while the other two species have smooth skull surfaces. Skull elongation and narrowing increased with decreasing body size from the larger *A. trachygaster*, via the medium-sized *A. seychellensis*, to the smaller *A. tachyscopaeus.* Analysis of body size-corrected 2D measurements of ten external morphological traits from 567 geckos revealed similar differences in head dimensions among the species, but also showed changes in mass and toe length. While we observed substantial morphological variation between islands, we did not find support for the previously identified northern and southern island group divergence within either *A. seychellensis* or *A. tachyscopaeus*. Nevertheless, we found strong support for the proposed divergence between northern and southern Mahé populations of *A. tachyscopaeus*, with variation in mass and head size between the two populations. We provide morphometric parameters to guide species identification within this gecko genus and highlight distinct population units that warrant further genomic investigation and prioritisation for conservation. Our findings illustrate the complexity of morphological diversification in archipelagos, where island-specific processes may outweigh regional biogeographic patterns. This underscores the need for integrative approaches to species delimitation and conservation in insular systems.

**Supplementary Information:**

The online version contains supplementary material available at 10.1186/s12862-026-02517-9.

## Background

The identification and formal recognition of species is central to understanding biodiversity patterns and change over time. Yet cryptic species, defined as evolutionarily distinct species with minimal morphological differences, often obscure the assessment of true diversity in terms of inventories and distributions [1]. This challenge is particularly pronounced on islands, where high endemism, geographic isolation, and conditions promoting rapid genetic divergence often act in concert to generate cryptic diversity [1]. Islands harbour a disproportionate share of global biodiversity [2], and understanding the evolutionary processes underlying their species richness, including the roles of vicariance, adaptive divergence, and genetic drift in shaping lineage diversity [3], depends on accurate species delimitation [4]. In addition, island taxa are often confined to restricted areas and small populations, and consequently highly vulnerable to extinction [5, 6] and population declines. Therefore, species-diversity knowledge on islands is essential for improving conservation assessments and offer valuable opportunities to investigate the macroevolutionary and biogeographic processes that generate biodiversity.

**Fig. 1:**
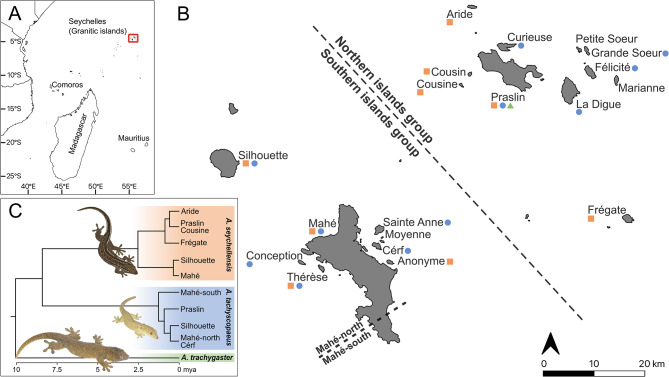
Granitic islands of the Seychelles archipelago. **A** – Location within the Western Indian Ocean. **B** – Map indicating the archipelagic division of the northern and southern island groups, as well as the north–south division of Mahé used in this study. Labelled islands represent study locations. Coloured symbols show confirmed species presence; orange square: *A. seychellensis*, blue circle: *A. tachyscopaeus*, and green triangle: *A. trachygaster*. **C** – Inter- and intraspecific phylogenetic relationship of *Ailuronyx* geckos, adapted from Rocha et al. [13]

The Seychelles archipelago lies in the Western Indian Ocean biodiversity hotspot. Its granitic islands, comprising a northern and a southern group (Fig. 1), are unique among oceanic islands, being composed of continental rock and having been isolated from mainland Gondwana since the Deccan volcanic event, approximately 65 Mya ago [7]. Because of their age and isolation from continental land masses, these islands support deeply divergent lineages of distinct species giving rise to exceptionally high endemism [8, 9]. However, the taxonomy of many Seychelles reptiles remains largely unresolved, partly due to phenotypic crypsis both within and among islands [10]. Despite the growing recognition of genetically distinct lineages within Seychelles reptiles [11–13], which mainly reflect geographical patterns separating northern islands from southern islands, formal taxonomic revisions of many reptile species are lacking, thereby delaying conservation assessments and downstream actions for numerous range-restricted endemics.

The Seychelles bronze geckos (Gekkonidae: *Ailuronyx*) exemplify this problem. Comprising ancient (Miocene), deeply divergent lineages, the genus consists of three currently recognised species; *A. seychellensis*, *A. tachyscopaeus*, and *A. trachygaster* that occur across the granitic islands of the archipelago [10]. Their species boundaries remain poorly studied and incompletely resolved, and phenotypic crypsis causes frequent misidentifications, impairing assessments of their distribution and conservation status [10]. Preliminary molecular findings on a small set of mitochondrial and nuclear genes suggest cryptic diversity within two of the species, *A. seychellensis* and *A. tachyscopaeus*, with notable divergences between the geographic region of the northern and southern granitic island groups and pronounced divergence within *A. tachyscopaeus* on the largest granitic island Mahé [13]. Furthermore, Rocha et al. [13] also found indications of morphological variation being congruent with their molecular data, but they refrained from making formal taxonomic changes in recognition of the need for more detailed molecular and morphological assessments.

Morphometric analyses are a valuable tool for species conservation. For example, they have enabled precise identification and clarification of taxonomy in mammals [14, 15], insects [16], and reptiles [17]. Morphometric analyses can also reveal morphological distinctiveness between native and invasive species, allowing for rapid in-situ discrimination between congeners [18], and aid in assessing the effects of anthropogenic pressures on animal populations [19–22]. Ultimately, identification of morphologically unique populations can reinforce conservation priority and lead to more effective conservation planning and action [23].

Morphometric analyses encompass two main approaches: traditional morphometrics, based on linear measurements between defined anatomical points [24], and geometric morphometrics, which uses landmark coordinates to capture shape variation while controlling for size, location, and orientation [25]. The geometric morphometrics approach is particularly useful for quantifying shape variation in features such as skeletal structures, because it preserves the geometric relationships among anatomical features [25]. In addition, a specimen’s morphology can be assessed by qualitatively describing shape, patterns, and structures, such as differences in bone surface sculpturing [26].

Here, we applied 3D and 2D morphometric analyses to investigate patterns of morphological divergence across the archipelagic range of the three *Ailuronyx* species. Specifically, we first describe general skull surface sculpturing of the three species. Secondly, to test hypotheses based on preliminary molecular findings reported by Rocha et al. [13], we evaluate interspecific, intraspecific, and within-island population-level differences in cranium and mandible shape, as well as in multivariate external morphometric traits. We expect that morphological differentiation in *Ailuronyx* mirrors the deep genetic divergences previously identified by Rocha et al. [13], hypothesising that (i) the three currently recognised species are morphologically distinct, (ii) geographically structured intraspecific divergence between northern and southern island groups is reflected in skull shape and external morphometric traits within *A. seychellensis* and *A. tachyscopaeus*, and (iii) population-level genetic divergence within *A. tachyscopaeus* on Mahé is accompanied by detectable morphological differentiation. Our goal is to provide information on morphometric traits that will improve species identification, distribution and conservation status of *Ailuronyx* geckos, while also providing a base for understanding evolutionary trajectories and adaptive processes of endemic island lineages.

## Methods

### Study site and data collection

The granitic islands of the Seychelles archipelago lie between 4°–5°S to 55°–56°E on the Mahé Plateau of the Seychelles Bank, a submerged microcontinent, in the Western Indian Ocean (Fig. 1A). Fieldwork was carried out between January 2024 and January 2025 across the entire distribution of the genus *Ailuronyx*, comprising 18 islands in the northern and southern granitic island groups (Fig. 1B), and all locations included in Rocha et al.’s [13] study (Fig. 1C). We used an opportunistic sampling strategy, surveying all accessible forested habitats without using predetermined transects or plots, and sampling all *Ailuronyx* spp. individuals encountered during day and night searches. Details of sampling per island, including sampling period, search effort, and number of collectors are provided in Table S1. We identified species in the field following Rocha et al. [13].

We found *A. seychellensis* on eight islands: Aride, Cousine, Frégate and Praslin in the northern island group, and Anonyme, Mahé, Silhouette and Thérèse in the southern island group (Fig. S1). This species also occurs on Cousin, part of the northern island group, which is represented in our data from museum specimens (see below). Previously reported records of this species from La Digue, Conception, Curieuse, Félicité, Marianne and Sainte Anne [10, and references therein, 27, and references therein] could not be confirmed for this species despite extensive searches on these islands.

We found *A. tachyscopaeus* on eleven islands, including Curieuse, Félicité, Grande Soeur, La Digue and Praslin in the northern island group, and Conception, Cérf, Mahé, Sainte Anne, Silhouette and Thérèse in the southern island group (Fig. S1). The occurrences on Sainte Anne and Thérèse represent the first records of this species on these islands [10]. We followed Rocha et al. [13] for the suggested population-level divergence on Mahé, where Mahé-south contains all geckos sampled in the southern-most Takamaka district (Fig. 1B).

We found *A. trachygaster* only on Praslin and did not find the species on Silhouette, despite five weeks of extensive searching, strengthening the conclusion of reports of the species there [28, 29] to be erroneous and the species to instead be endemic to Praslin [13, 30] (Fig. S1).

### Three-dimensional dataset

To investigate skull surface sculpturing and assess cranium and mandible shape, we collected eight *A. seychellensis* and 13 *A. tachyscopaeus* voucher specimens across their archipelagic range. We included an additional nine *A. seychellensis* and three *A. tachyscopaeus* specimens with precise locality data, i.e. GPS coordinates to island level accuracy, in our study from the collections of the Zoological Research Museum Alexander Koenig, Bonn, Germany (specimen catalogue numbers with ZFMK prefix), and Natural History Museum London, UK (BMNH), respectively (Table S2). Considering the critically endangered status of the Praslin endemic *A. trachygaster* [30], we did not collect voucher specimens of this species in the field. However, because of a recent appearance of this species in the international pet trade [30], we sourced nine *A. trachygaster* voucher specimens by collecting deceased animals from private collections. We stored five *A. trachygaster* specimens at the University of Porto (CIBIO) and all remaining vouchers were deposited at the ZFMK (catalogue numbers: ZFMK-HERP-104218 to 104242, Table S2).

We performed high-resolution X-ray micro-computed tomography (µCT) scanning using a YXLON FF20 CT scanner at the Morphology Lab of the Leibniz Institute for the Analysis of Biodiversity Change (LIB) in Hamburg, Germany. Three specimens were scanned on a Nikon Metrology HMX ST 225 microCT at the Imaging and Analysis Centre of the National History Museum, London, UK, and one µCT scan was obtained from MorphoSource (CAS: HERP:167549, ark:/87602/m4/M101124). Alcohol-preserved specimens were tightly wrapped in soft artificial wadding, mounted in plastic cylinders, and scanned in an ethanol saturated environment. Sexual maturity was determined by examining the presence of endolymphatic calcium sacs, femoral pores and hemipenal bulges. Individuals exhibiting the latter two characteristics were classified as males, which was further confirmed by the detection of hemibacula in µCT scans. We excluded the juvenile specimen ZFMK-HERP-48662 from analyses to avoid possible ontogenetic effects on shape due to incomplete fusion of the skull elements [25]. We refrained from excluding other smaller specimens due to their fused skull elements and signs of sexual maturity. Information on the specimens included in this study and parameters used for scanning are summarised in Table S2.

The 3D articulated skull volumes were segmented using Amira v.6.0.1 (Thermo Fisher Scientific). Following Engelkes [31], two methods to define segmentation thresholds were compared for one arbitrarily selected CT volume per species. First, Otsu’s local threshold cluster algorithm was applied with a local domain radius of 5 pixels using the Fiji [32, based on ImageJ v.1.54, 33] plugin ‘*Auto Local Threshold’.* For each CT volume, the Otsu algorithm was run on three different version of the dataset: The original images and two resliced stacks (reslicing directions: top to bottom, left to right). The three thresholding results of a given volume were then combined using the ‘*Arithmetic’* function in Amira with a ‘2of3’ voting rule, where a given voxel was classified as bone if it was recognised as bone in any two of the three thresholded volumes [34]. Second, we calculated a global threshold in Amira through the following steps: (i) shrinking of roughly segmented background area by 30 voxels and roughly segmented bone by 10 voxels; (ii) calculation of mean grey values for bone and background segmentations using the ‘*MaterialStatistics’* function; and (iii) calculation of the optimal global threshold [i.e. half maximum height, 35] using the formula $\:\frac{a-b}{2}+b$, where a = mean bone grey value and b = mean background grey value. Then, we overlayed the segmentation results of the Otsu and global threshold methods in the segmentation panel of Amira and visually checked for differences. Additionally, we also generated surface meshes of both the Otsu and global threshold segmentations using a customized version of the ‘*multiExport’* script [36; custom modification: usage of ‘Generate Lego Surface’ instead of ‘Isosurface’ in combination with ‘Extract surface’] in Amira. The surfaces were simplified (i.e. reduction of polygon count and smoothing) to a subjectively optimal degree and the Hausdorff Distances between surfaces was calculated in MeshLab v.2023.12 [37]. We found minimal differences in the region of 1–2 voxels between the two segmentation approaches (Fig. S2) and therefore chose the less labour-intense global thresholding to segment all CT volumes.

For each CT volume, the optimal global threshold was calculated, and the segmentation was performed using the ‘*magic wand’* tool in Amira. The cranium was separated from the mandible and the first vertebra using the ‘*brush*’ tool. Separate simplified surfaces of the cranium and the mandible were exported as above.

We used the ‘*markup’* module in 3D Slicer v.5.8.1 [38] to digitize 3D landmarks. Landmark locations were chosen based on previous studies of geckos [39, 40] and landmarks were placed by the same observer (MAR) to avoid observer-dependent bias. A total of 120 and 48 fixed landmarks were placed on homologous positions on the cranium and mandible, respectively (Fig. S3, Table S3). For the cranium, 54 landmarks were placed symmetrically each on the left and right side and 12 landmarks along the mid-sagittal plane. For the mandible, 24 landmarks were placed on each side. One specimen of each species was landmarked three times to assess observer related error in landmark placing. These specimens clustered together more closely than other specimens of the same species, indicating negligible landmarking error (Fig. S4).

The final 3D dataset comprised landmarks on the crania and mandibles of 42 geckos, including 17 *A. seychellensis* (nine islands), 16 *A. tachyscopaeus* (11 islands) and nine *A. trachygaster*. We investigated skull surface sculpturing visually following [26] and assessed cranium and mandible shape statistically as described below.

### Two-dimensional dataset

**Fig. 2:**
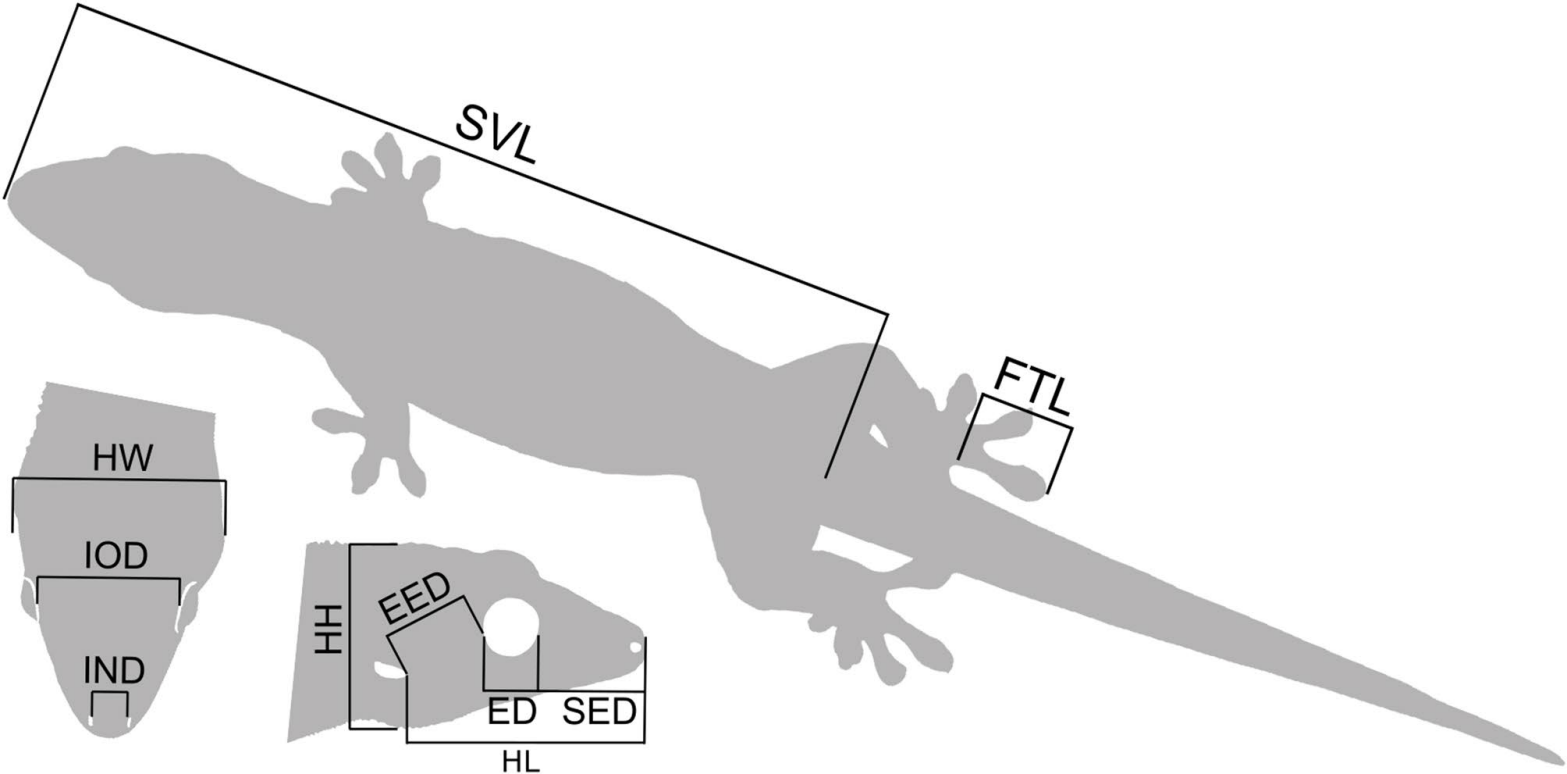
Illustration of external two-dimensional multivariate morphometric measurements taken from each gecko

To assess external body morphometrics, a total of 627 geckos across 15 islands were captured and measured. Head dimensions are widely used in lizard taxonomy and are functionally linked to bite performance, feeding ecology, and sexual selection, making them informative traits for detecting both interspecific and intraspecific divergence and adaptation [41]. Snout and orbital measurements capture variation in facial proportions that frequently differ among closely related species and populations [13, 42]. The following morphometric measurements were taken to the nearest 0.01 mm with callipers (Fig. 2): eye diameter (ED), eye-ear distance (EED), fourth toe length (FTL), head height (HH), head length (HL), head width (HW), internarial distance (IND), interorbital distance (IOD) and snout-eye distance (SED). Snout-vent length (SVL) was measured to the nearest 1 mm with a ruler and mass (Weight) was measured to the nearest 0.5 g using a Pesola precision scale. Bilateral symmetrical measurements were taken on the right side of the geckos (Fig. 2), and all measurements were taken by the same observer (MAR). Sex was determined by examining the presence of femoral pores and hemipenal bulges, with individuals exhibiting these characters classified as males. No published data on minimum size at sexual maturity exist for any species of the genus *Ailuronyx*. We therefore used a conservative approach to exclude presumed juveniles (i.e., excluding small individuals lacking any signs of sexual maturity [43, 44]) from the data to avoid possible ontogenetic allometric shifts in body dimensions [45, 46]. In addition, we also excluded specimens with missing data. We acknowledge that the absence of visible secondary sexual characters does not exclude the possibility that some individuals may have been sexually mature but undetectable as such based on external examination alone, particularly in females. However, we repeated all 2D–analyses including the presumed juveniles and found overall results and conclusions remained unchanged (Table S4), indicating that our findings are robust to uncertainty in maturity classification. The final 2D dataset consisted of 567 geckos including 246 *A. seychellensis* (eight islands), 206 *A. tachyscopaeus* (11 islands) and 115 *A. trachygaster*. Measured geckos were released at the exact location of capture.

### Data analyses

All analyses were run in R v.4.5.0 [47] via RStudio v.2024.12.1.563 [48]. We used the R packages *abind* v.1.4-8 [49], *readr* v.2.1.5 [50], *dplyr* v.1.1.4 [51], *stringr* v. 1.5.1 [52], *tidyr* v.1.3.1 [53] and *forcats* v.1.0.0 [54] for data wrangling, and *ggplot2* v.3.5.2 [55], *ggforce* v.0.5.0 [56], *ggpubr* v.0.6.0 [57], and *svglite* v.2.2.1 [58] for visualisation. All datasets were analysed for both species-level and intraspecific regional differences.

Three-dimensional data were analysed using the R package *geomorph* v.4.0.10 [59, 60] with its dependencies *RRPP* v.2.1.2 [61, 62], *rgl* v.1.3.18 [63] and *Matrix* v.1.7-3 [64]. All analyses were performed separately for crania and mandibles. Missing landmark data (total of 14 cranial and 3 mandibular landmarks across the dataset) were estimated using a within-species thin-plate spline method, which interpolates landmarks on a reference specimen (in this study: species Procrustes mean shape) to estimate the locations of missing landmarks on a target specimen [65]. For this, the ‘*estimate.missing’* function was applied to species-specific subsets of the data. We obtained shape variables by performing a generalized Procrustes analysis [66] using the ‘*gpagen’* function, which removes scale, location, and orientation by scaling to unit centroid size (CS), translation, and rotation. Because skulls are symmetric along the mid-sagittal plane, we extracted the bilateral symmetry component of shapes using the ‘*bilat.symmetry’* function and used the resulting landmark data for downstream analyses. We used permutational multivariate ANOVAs to test for significant shape differences between species and within mtDNA-informed intraspecific geographical patterns using the ‘*procD.lm’* function with 9999 permutations. Since the allometric effect of CS on shape was significant (tested in a separate model using the ’*procD.lm’* function), we included the log-transformed CS in all models as a main effect [67]. Similarly, to account for sexual shape dimorphism, we included sex as a main effect in all models, as well as the interaction between sex and species. Thus, we tested whether inter- and intraspecific variation in shape is independent of size and sex. Due to sample size limitations in the species-specific datasets, with most islands represented by a single specimen, we did not include island as a nested factor within regions in these analyses. Post hoc pairwise comparison using the ‘*pariwise’* function was used to determine species-level differences in shape by providing a null model fit without species to exclude covariate effects on species [61]. We used principal component analyses (PCAs) and boxplots to visualise shape variation among different species and intraspecific geographic patterns. To better visualise shape variation along the first two PC axes we used a three-dimensional warping approach using the ‘*warpRefMesh’* and ‘*plotRefToTarget*’ functions.

Two-dimensional data were analysed in a similar way to the 3D data. All analyses were performed separately for the full and species-specific datasets, respectively. Morphological traits are often affected by allometric size effects [68]. Therefore, we first adjusted morphometric variables (ED, EED, FTL, HH, HL, HW, IND, IOD, SED, Weight) to remove the effect of body size, using snout-vent length (SVL) as the size metric, following Roesch et al. [69]. For each variable, we applied the transformation $\:Z={{Y}_{i}(\stackrel{-}{SVL}\:/\:{SVL}_{i})}^{b}$, where Z represents the transformed, size-corrected, value, $\:{Y}_{i}$ is the raw measurement for individual *i*. $\:\stackrel{-}{SVL}$ is the mean SVL across all individuals in the respective dataset, $\:{SVL}_{i}$ is the SVL of individual *i*, and *b* is the global slope of the linear regression between log(Y) and log(SVL) across all species and sexes in the respective dataset. This transformation scales individuals to the same size and adjusts their shape according to allometry [68], yielding size-corrected values that were used in all subsequent analyses in place of raw measurements. For interspecific analyses, the slope *b* was estimated globally across all species and sexes. For intraspecific analyses, the slope *b* was re-estimated separately using only conspecific individuals to account for potentially different allometric trajectories among species. Snout-vent length was excluded as a variable from the corrected dataset as it served as the size metric. Body size-corrected response variables were then scaled and converted into a matrix. We used linear models with a randomized residual permutation procedure using the ‘*lm.rrpp’* function from the R package *RRPP* with 9999 permutations. We included sex as a main effect, and its interaction with species, in the models, which allowed us to assess the presence of sexual size dimorphism in intraspecific models and account for it in interspecific and geographic models. We included islands as a fixed effect in the interspecific model, and as a factor nested within regions in the species-specific analyses to account for the non-independence among individuals within islands. Post hoc pairwise comparison using the ‘*pairwise’* function was used to determine species-level differences in body size-corrected multivariate measurements by providing a null model fit without species to exclude covariate effects on species [61]. We used PCAs to visualise shape variation among different species and intraspecific geographic patterns.

## Results

### Skull surface sculpturing

**Fig. 3:**
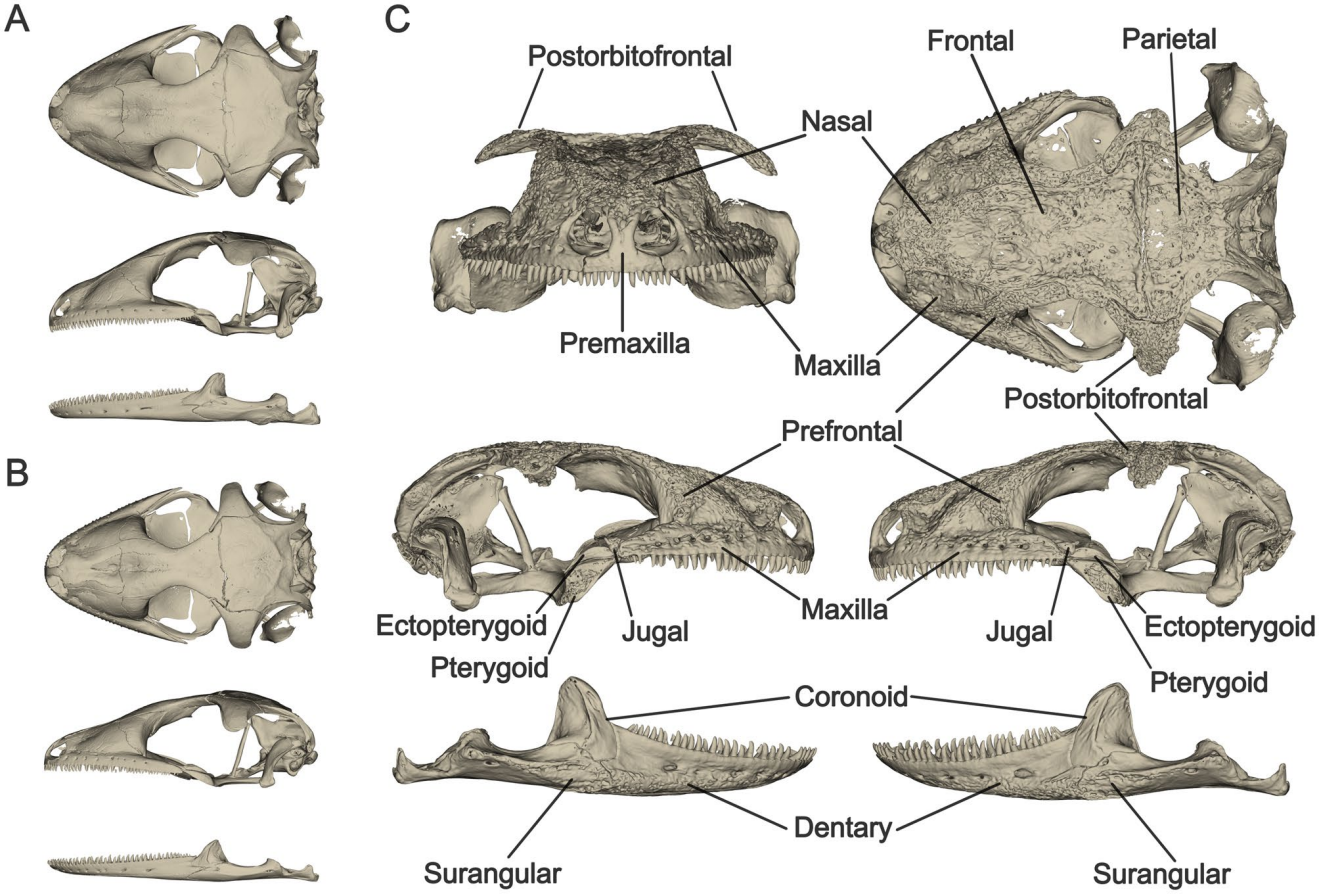
**A** – Smooth skull surfaces on cranium and mandible of *Ailuronyx seychellensis* (ZFMK-HERP-104218). **B** – Smooth skull surfaces on cranium and mandible of *A. tachyscopaeus* (ZFMK-HERP-104224). **C** – Rugose structuring on the cranium and mandible of *A. trachygaster* (HK-TRA-04). Only bones with structuring are labelled

Visual assessment of the generated 3D surfaces showed pronounced differences in skull surface sculpturing between the three species. *Ailuronyx seychellensis* and *A. tachyscopaeus* both presented smooth skull surfaces (Fig. 3A, B). In contrast, *A. trachygaster* exhibited rugose sculpturing on up to ten cranial bones, including the premaxilla, maxilla, nasal, prefrontal, frontal, parietal, postorbitofrontal, pterygoid, ectopterygoid, and jugal. Rugose sculpturing was also present on the dentary, coronoid and surangular of the mandible (Fig. 3C).

**Fig. 4:**
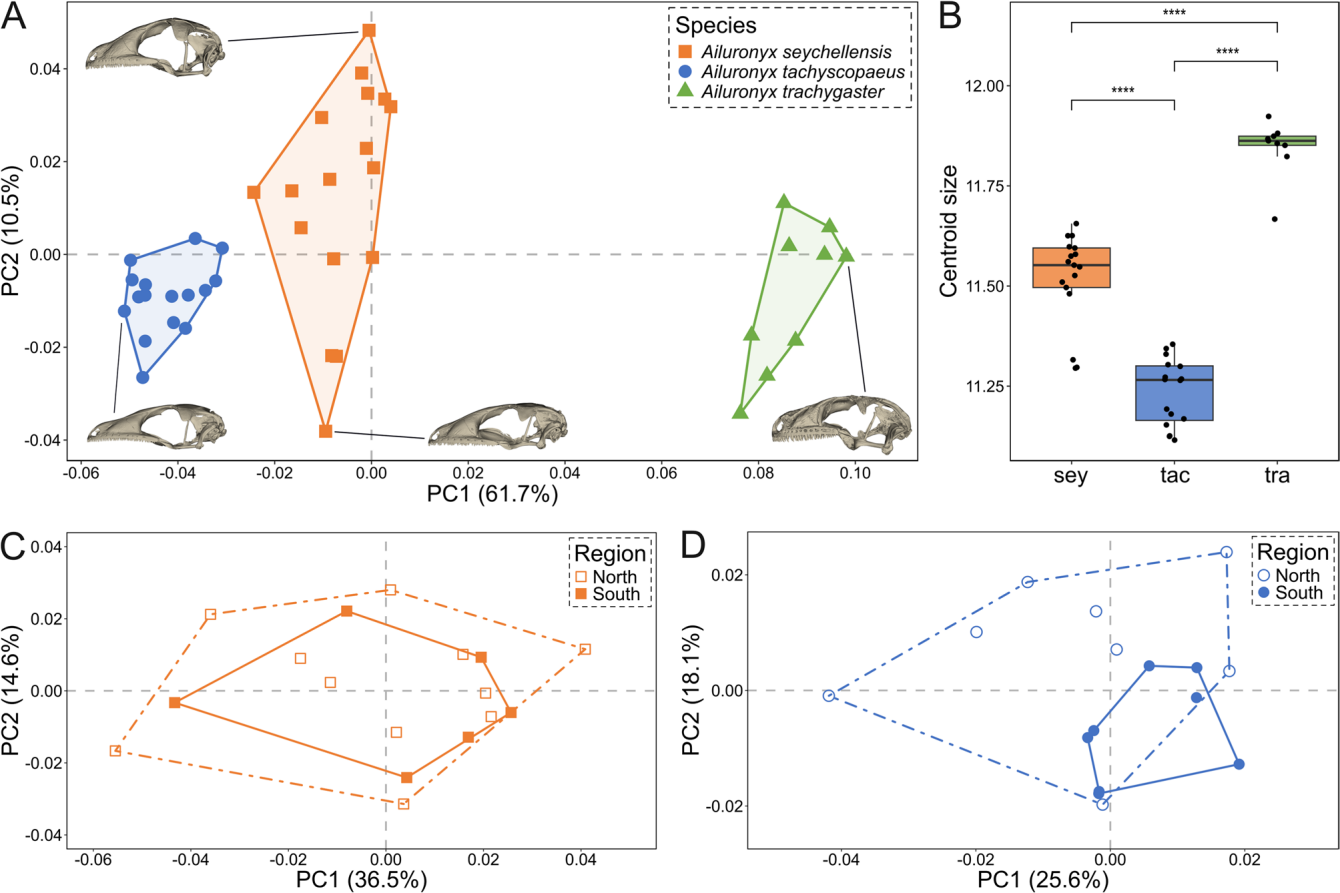
**A** – First two principal components of cranial shape with regard to *Ailuronyx* species (based on 120 fixed landmarks). **B** – Difference in centroid size between species. sey = *A. seychellensis*, tac = *A. tachyscopaeus* and tra = *A. trachygaster*. Four asterisks (****) indicate a significance level of *p* < 0.001. **C** – Cranial shape of *A. seychellensis* with regard to northern and southern island groups. **D** – Cranial shape of *A. tachyscopaeus* with regard to northern and southern island groups

### 3D geometric morphometrics

#### Cranium morphology

There was strong cranial shape variation among the species, with the first two principal components accounting for 72.3% of the total variation (Fig. 4A). PC1 represented 61.7% of the cranial variation, with one extreme (high negative values) being skulls with long, flattened, dorsally tilted snouts and a narrow, low posterior cranium, and the other extreme (high positive values) being laterally flared, dorsally elevated posterior cranium (Fig. S5A, B). PC2 represented 10.5% of the cranial variation, mainly representing changes in cranial height. The second component separated skulls with more narrow, shallow snouts from those with broader, deeper snouts, whereas only minor shape differences occur in the posterior part of the cranium (Fig. S5C, D). Multivariate regression of shape revealed allometric differences between species (*Z* = 5.2459, *p* < 0.001, *R²* = 0.1797; Fig. 4B, Table S5). Pairwise comparison confirmed differences among all species (*p* < 0.001; Table 1), with the greatest shape disparity occurring between *A. tachyscopaeus* and *A. trachygaster*, reflecting moderate differences in snout length and cranium vaulting. Intraspecific shape differences were not significant between northern and southern island groups within each of the two tested species, *A. seychellensis* (*Z* = 0.6875, *p* = 0.2425, *R²* = 0.05992; Fig. 4C, Table S5) and *A. tachyscopaeus* (*Z* = 1.60611, *p* = 0.0598, *R²* = 0.10088; Fig. 4D, Table S5).

#### Mandible morphology

**Fig. 5:**
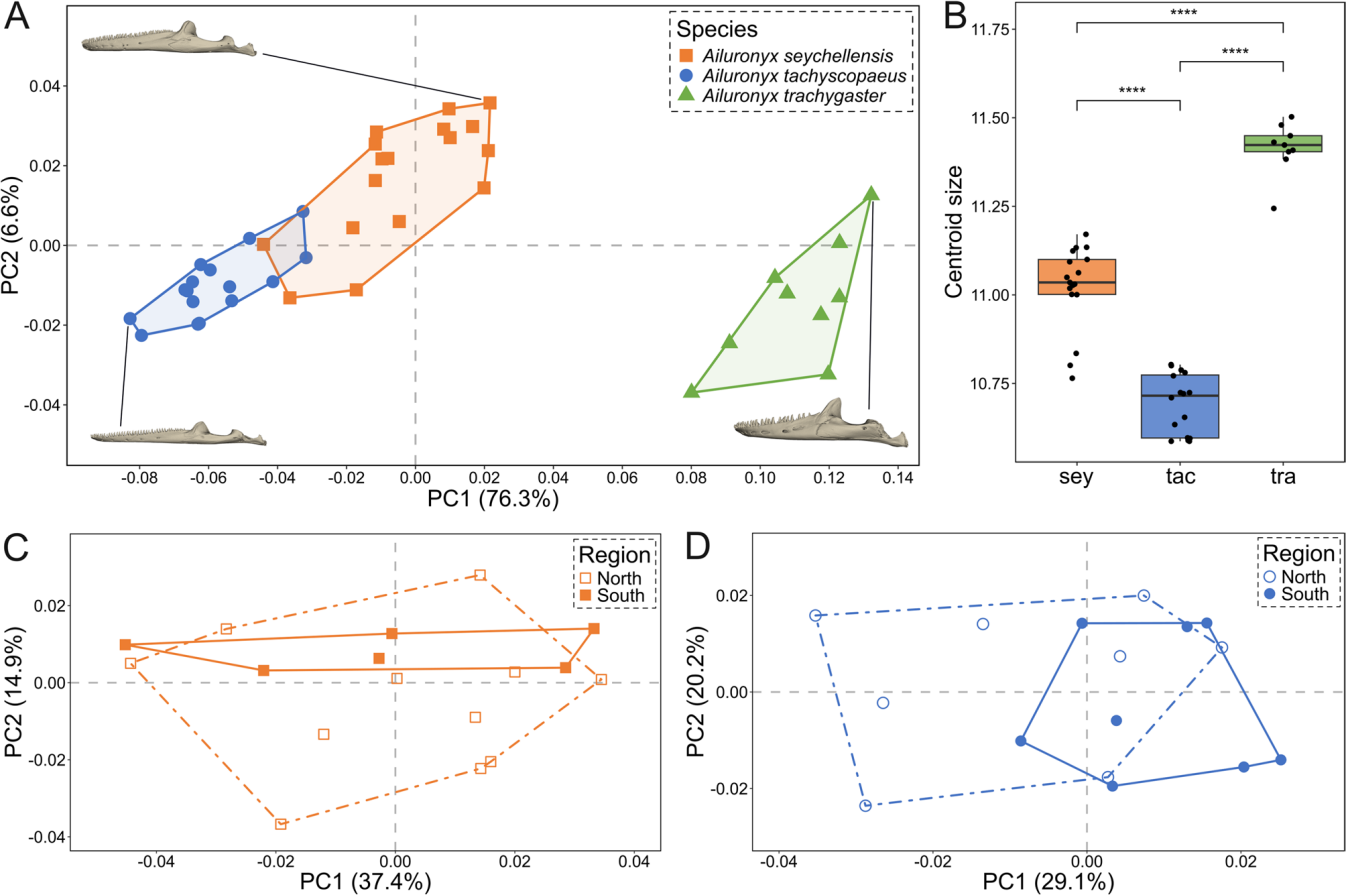
**A** – First two principal components of mandible shape with regard to *Ailuronyx* species (based on 48 fixed landmarks). **B** – Difference in centroid size between species. sey = *A. seychellensis*, tac = *A. tachyscopaeus* and tra = *A. trachygaster*. Four asterisks (****) indicate a significance level of *p* < 0.001. **C** – Mandible shape of *A. seychellensis* with regard to northern and southern island groups. **D** – Mandible shape of *A. tachyscopaeus* with regard to northern and southern island groups

Mandible shape varied between the three species with the first two principal components accounting for 82.9% of the total variation (Fig. 5A). PC1 explained 76.3% of the mandibular shape variation, with one extreme (high negative values) having low-coronoid, elongated mandibles and the other extreme (high positive values) having shorter, high-coronoid mandibles. Additionally, there is a positional reconfiguration of the retroarticular process along PC1 (Fig. S5E, F). PC2 explained 6.6% of the mandibular variation, primarily reflecting changes in the ventral curvature of the dentary, and changes in the mediolateral flaring of the rami, which varied from narrower, less flared rami to laterally flared rami (Fig. S5G, H). Multivariate regression of shape revealed allometric differences between species (*Z* = 4.5637, *p* < 0.001, *R²* = 0.13635; Fig. 5B, Table S5). Pairwise comparison confirmed significant mandibular shape differences among all species (*p* < 0.001; Table 1). The greatest shape disparity occurred between *A. tachyscopaeus* and *A. trachygaster*, reflecting moderate differences in mandibular elongation and coronoid height. Intraspecific differences were not significant between northern and southern island groups within both *A. seychellensis* (*Z* = 0.29259, *p* = 0.3838, *R²* = 0.05682; Fig. 5C, Table S5) and *A. tachyscopaeus* (*Z* = 1.4854, *p* = 0.0741, *R²* = 0.10610; Fig. 5D, Table S5).

### 2D morphometrics

**Fig. 6:**
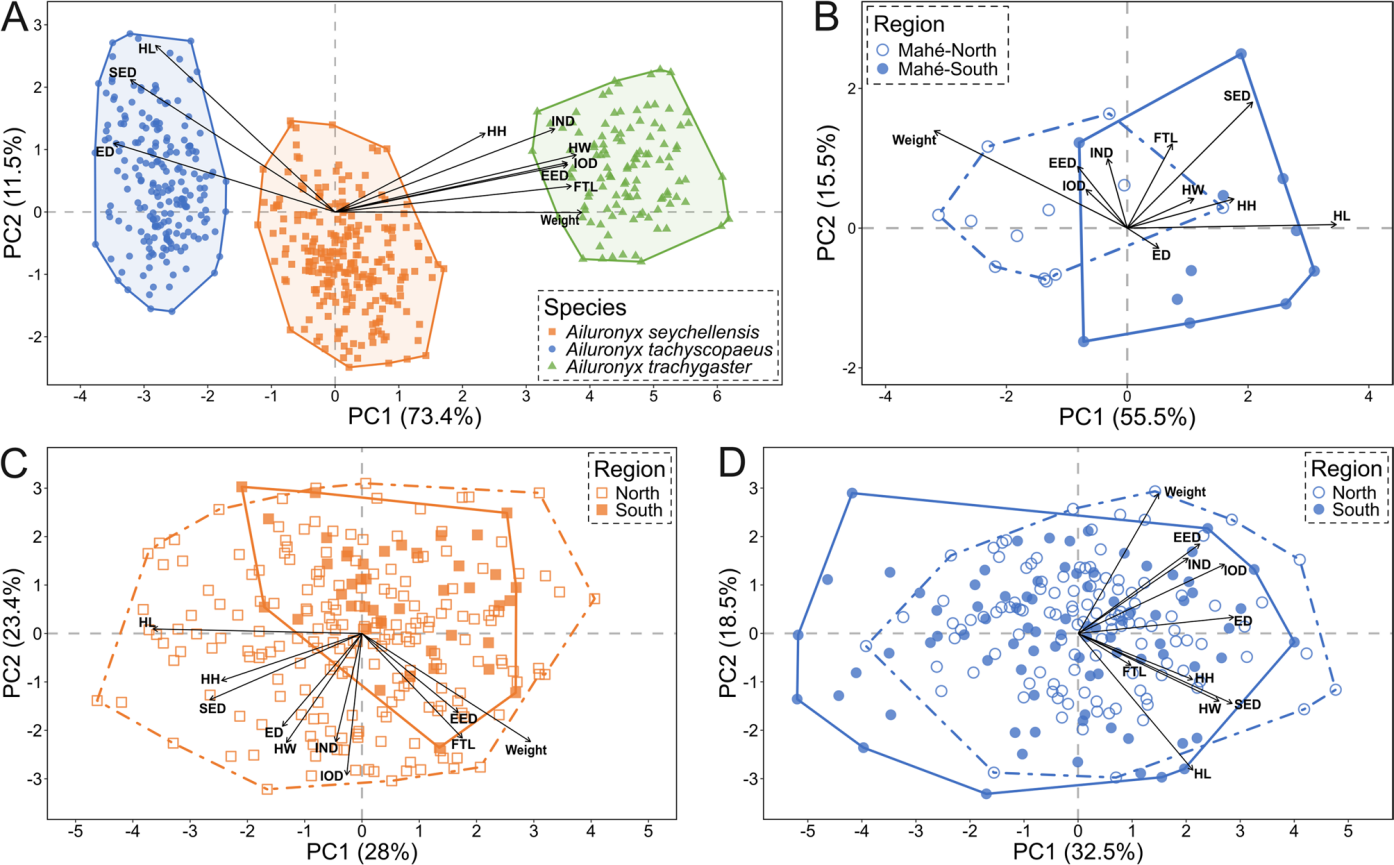
Principal Component Analysis of body-size corrected multivariate morphometric traits. **A** – between species, **B** – within *A. tachyscopaeus* Mahé-north and Mahé-south populations, **C** – within *A. seychellensis* northern and southern island groups, and **D** – within *A. tachyscopaeus* northern and southern island groups. Morphometric trait abbreviations used in the figure panel are eye diameter (ED), eye-ear distance (EED), fourth toe length (FTL), head height (HH), head length (HL), head width (HW), internarial distance (IND), interorbital distance (IOD) and snout-eye distance (SED)

Body size-corrected external morphometric traits varied between the three species, with the first two principal components accounting for 84.9% of the total variation (Fig. 6A). PC1 explained 73.4% of the variation, separating the three species into distinct clusters. *Ailuronyx trachygaster* on the one extreme (high positive values) differed from all other species by having grater mass, longer toes and overall wider and higher heads (|loadings|: EED = 0.91, FTL = 0.93, HH = 0.59, HW = 0.95, IND = 0.86, IOD = 0.91, Weight = 0.97). *Ailuronyx tachyscopaeus* on the other extreme (high negative values) differed from all other species by having longer snouts, overall elongated heads and larger eyes (|loadings|: ED = 0.87, HL = 0.71, SED = 0.80). PC2 explained 11.5% of the variation with head length having the largest effect (|loadings|: HL = 0.67, SED = 0.53; all others < 0.4). Multivariate regression revealed significant differences between species (*Z* = 11.7334, *p* < 0.001, *R²* = 0.76739; Table S5). Pairwise comparison confirmed significant differences among all species (*p* < 0.001), with the greatest disparity occurring between *A. tachyscopaeus* and *A. trachygaster* (Euclidean distance = 7.344258), reflecting strong multivariate separation (Table 1).

**Table 1:** Pairwise comparisons of shape variation between species

	d	UCL (95%)	Z	Pr > d
**3D geometric morphometrics**				
*Cranium*				
sey: tac	0.06641	0.04764	2.90547	**< 0.001**
sey: tra	0.10657	0.05754	3.94822	**< 0.001**
tac: tra	0.15908	0.08125	3.94816	**< 0.001**
*Mandible*				
sey: tac	0.08305	0.05616	2.91747	**< 0.001**
sey: tra	0.14150	0.07177	3.48124	**< 0.001**
tac: tra	0.21344	0.11163	3.21630	**< 0.001**
**2D morphometrics**				
sey: tac	3.28277	2.24543	7.63308	**< 0.001**
sey: tra	4.75194	2.54060	12.35258	**< 0.001**
tac: tra	7.34426	4.10140	17.28796	**< 0.001**

sey = *Ailuronyx seychellensis*, tac = *A. tachyscopaeus*, tra = *A. trachygaster*, d = observed distance between group means, UCL (95%) = upper 95% confidence limit of the distance under permutation, Z = effect size, and Pr > d = permutation p-value

Intraspecific comparisons of *A. seychellensis* revealed no significant differences in size-corrected external body dimensions between northern and southern island groups (*Z* = -0.5512, *p* = 0.7096, *R²* = 0.02761; Table S5), whereas variation among islands within regions was substantial (*Z* = 13.0758, *p* < 0.001, *R²* = 0.2564, Table S5). The first two principal components accounted for 51.4% of the total variation (Fig. 6C). PC1 explained 28% of the variation and was characterised by strong positive loadings of EED = 0.41, FTL = 0.44 and Weight = 0.74, and strong negative loadings of HH = -0.61, HL = -0.91 and SED = -0.66. PC2 explained 23.4% of the variation, with strong negative loadings of EED, ED, FTL, HW, IND, IOD, and Weight, which were all smaller than − 0.40.

Intraspecific comparisons of *A. tachyscopaeus* revealed no significant differences in size-corrected external body dimensions between northern and southern island groups (*Z* = 0.2808, *p* = 0.3875, *R²* = 0.02489; Table S5), whereas variation among islands within regions was substantial (*Z* = 10.2101, *p* < 0.001, *R²* = 0.2343, Table S5). The first two axes of the PCA accounted for 51% of the total variation (Fig. 6D). PC1 explained 32.5% of the variation and was characterised by strong positive loadings of EED, ED, HH, HL, HW, IND, IOD, SED, which were all larger than 0.4. PC2 explained 18.5% of the variation, with strong positive loadings of EED = 0.46 and Weight = 0.72, and a strong negative loading of HL = -0.71.

Within the largest granitic island (Mahé), the population-level comparisons of *A. tachyscopaeus* revealed significant differences in size-corrected external body dimensions between the north and the south of the island (*Z* = 3.2173, *p* < 0.001, *R²* = 0.26504; Table S5). The first two axes of the PCA accounted for 71% of the variation (Fig. 6B). PC1 explained 55.5% of the variation. *Ailuronyx tachyscopaeus* specimens from Mahé-south differ from the Mahé-north population by having proportionally longer snouts and overall larger, elongated heads (|loadings|: SED = 0.69, HH = 0.59), and a lighter body mass (|loading|: Weight = 1.06). PC2 explained 15.5% of the variation, mostly showing within population variation, with positive loadings of FTL = 0.40, SED = 0.60 and Weight = 0.46.

Significant, but modest differences in size-corrected external body dimensions between sexes were found in all three species; *A. seychellensis* (*Z* = 4.8177, *p* < 0.001, *R²* = 0.03561; Tables S5, S6, Fig. S6), *A. tachyscopaeus* (*Z* = 3.9481, *p* < 0.001, *R²* = 0.03092; Tables S5, S6, Fig. S6), and *A. trachygaster* (*Z* = 5.816, *p* < 0.001, *R²* = 0.07956; Tables S5, S6, Fig. S6). In *A. seychellensis*, females were characterised by relatively longer fourth toes (FTL), larger eye-ear distances (EED) and greater mass, while males had relatively longer heads (HL), longer snout-eye distances (SED) and higher heads (HH). In *A. tachyscopaeus*, females had greater mass, while males were characterised by relatively longer and larger heads (HH, HL, HW, SED). In *A. trachygaster*, females were characterised by wider and broader heads (EED, HH, HW, IND, IOD), and greater mass, while males had relatively longer snout-eye-distances (SED) and longer heads (HL).

## Discussion

Our 3D and 2D morphometric analyses reveal distinct patterns of cranial, mandibular and external morphometric trait variation between the three currently recognised species of Seychelles *Ailuronyx* geckos. We found pronounced and significant interspecific differences, including a distinct rugose skull sculpturing in *A. trachygaster* compared to the smooth skulls of *A. seychellensis* and *A. tachyscopaeus*. Skull elongation and narrowing increased with decreasing body size from the larger *A. trachygaster*, via the medium-sized *A. seychellensis*, to the smaller *A. tachyscopaeus*. Similarly, we also observed a decrease in weight and toe length with decreasing body size between the three species. Importantly, our results reveal that intraspecific differences in size-corrected external body dimensions occur primarily at the island-level rather than reflecting a broad geographic pattern between northern and southern island groups of *A. seychellensis* and *A. tachyscopaeus*. However, within the largest island of Mahé, the Mahé-south population of *A. tachyscopaeus* exhibited significantly larger heads and lower body weight compared to the Mahé-north population. Overall, our findings provide evidence of both inter- and intraspecific morphological divergences in Seychelles *Ailuronyx* geckos, with implications for understanding the evolutionary processes shaping diversity across this ancient archipelago.

Bone surface structuring has been observed in various degrees on the cranial elements of many vertebrate groups [70–72]. Although most geckos have smooth skull surfaces, several taxa across the gekkotan phylogeny display some sort of sculpturing (i.e., grooved, pitted, rugose) [26]. However, only five species of geckos are known to exhibit sculpturing across more than seven skull bones, with *Chondrodactylus bibronii* being the most extreme to date, exhibiting pronounced pitted sculpturing that is present on ten bones [26]. Our results show that *A. trachygaster* exhibits an extreme case of skull sculpturing among geckos, with rugose sculptures on as many as 13 skull bones. This contrasts with its sister species *A. seychellensis* and *A. tachyscopaeus*, both of which have smooth skull surfaces. Intraspecific patterns in sculpturing intensity in *A. trachygaster* could indicate ontogenetic variation, as documented in other gecko species [26], and merits further investigation. Bone sculpturing may play a role in thermoregulation [71], gland excretion [73], or visual signalling [74]. The extent of bone sculpturing in *A. trachygaster* is unique among geckos [26] and raises broader questions about the evolutionary drivers of cranial ornamentation in lizards, including whether such traits arise through ecological adaptation, sexual selection, physiological adaptations, or phylogenetic constraint, and what functional role they may serve in island-endemic lineages with specialised ecologies.

Vertebrate skulls reflect complex patterns of evolutionary pressures, including developmental constraints, ecological adaptation and size-related allometry [e.g., 75–77]. The craniofacial evolutionary allometry (CREA) hypothesis proposes that larger species tend to evolve longer faces, a pattern supported in numerous taxa including mammals [78], birds [79] and reptiles [40, 80]. Intriguingly, *Ailuronyx* geckos may deviate from this pattern, with the largest gecko in our study, *A. trachygaster*, presenting, after size correction, a shorter snout compared to the smaller species. However, our analyses treated allometry as a covariate rather than explicitly testing CREA predictions, and therefore this observation should be considered preliminary. If confirmed, such deviation could reflect adaptive diversification, as observed in cases of likely paedomorphic skulls, such as in fossorial and arid-adapted lizards [81, 82], as well as in the specialized skull configurations in snakes [83]. Functionally, shorter, higher heads and deeper mandibles in lizards are repeatedly linked to greater bite forces and shifts toward harder or larger prey and/or different microhabitats [77, 84–86]. We hypothesise that ecological adaptation may underlie the shape differences observed in *Ailuronyx*, particularly given that *A. trachygaster* is known to be a palm specialist forming a close association with the coco de mer (*Lodoicea maldivica*) and feeding primarily on the palm’s pollen [30, 44]. Future studies that explicitly test allometric scaling of cranial shape across *Ailuronyx* species, combined with dietary assessments, would clarify whether the patterns observed here represent true departures from CREA and whether they are linked to ecological divergence.

In the Seychelles, limited dispersal and frequent sea-level oscillations combined with the ancient granitic formation of the inner islands have strongly shaped the genetic structure of the herpetofauna [8, 9, 11–13, 87, 88]. In *Ailuronyx* geckos, previous molecular analyses identified ancient, deeply divergent lineages with evidence of possible unresolved species boundaries within two of the species, *A. seychellensis* and *A. tachyscopaeus* [13]. For these two species, there was an indication of pronounced mtDNA-sequence divergences between the northern and southern island groups (more pronounced in *A. seychellensis*) and additional divergence within *A. tachyscopaeus* on Mahé [13]. Using geometric morphometrics for cranial and mandibular shape, we found clear, significant differences between the currently recognised species, but could not detect intraspecific differentiation corresponding to the hypothesised northern and southern geographic groups. Similarly, our analysis of body size-corrected multivariate 2D morphometric measurements did not support regional divergence within either *A. seychellensis* or *A. tachyscopaeus*. When accounting for the nested structure of individuals within islands, regional differences were not significant, whereas island-level variation was substantial. This indicates that morphological differentiation in these species does not follow a simple north-south gradient, but rather reflects fine-scale, island-specific evolutionary trajectories. In contrast, we found strong evidence for population-level divergence within *A. tachyscopaeus* on Mahé, with specimens from the southern tip of the island (Takamaka district) differing significantly from northern Mahé populations, corroborating the distinct lineage proposed by Rocha et al. [13]. The mismatch between genetic and morphological structuring at the regional level, combined with pronounced island-level and within-island divergence, suggests that morphological evolution in *Ailuronyx* is shaped by local ecological conditions and demographic history rather than by broad-scale vicariance alone. Hence, our findings partly support previous evidence for intraspecific structure, while highlighting that morphological divergence within *Ailuronyx* species operates primarily at the island-level scale rather than across broad geographic regions. Such fine-scale, island-specific divergence is consistent with patterns observed in other archipelago-distributed taxa [89], where limited inter-island dispersal and heterogeneous local environments can drive independent morphological trajectories on individual islands [3], and underscores the importance of treating island populations as potentially independent evolutionary units. The substantial among-island variation in *Ailuronyx*, therefore, warrants further investigation and represents an important contribution to an integrative taxonomic revision of the genus.

Prior work on Seychelles geckos shows contrasting patterns of morphological divergence [42, 87, 90]. Studies on *Phelsuma* day geckos from the granitic islands reported that their body size and external morphology diverged idiosyncratically across regions and lineages. These differences seem to be linked to the availability of distinct habitats and resources on each island, rather than to character displacement relative to coexistence time [87]. On the other hand, a study on the gecko genus *Urocotyledon* (sucker-tailed geckos) showed very little morphometric divergence between two closely related species, *U. norzilensis* found in the northern granitic islands and its sister species *U. inexpectata* in the southern granitic islands [42, 90]. Given these contrasting morphological patterns found, and the recurring history of isolation, colonization, and potential secondary contact events across Seychelles, a range wide genomic assessment of *Ailuronyx* geckos is needed to infer species boundaries and to disentangle putative complex evolutionary scenarios, such as ancient hybridization and/or ongoing gene flow and adaptation. Understanding how these processes interact with the morphological patterns documented here will be essential for resolving whether the observed island-level divergences represent early stages of speciation, phenotypic plasticity in response to local conditions, or neutral morphological drift in small, isolated populations.

Sexual size dimorphism was significant but modest in all three *Ailuronyx* species, with the sexes differing mainly in head size and body mass. This pattern aligns with a general trend among geckos, although sexual dimorphism remains understudied compared to other lizard families [91]. Across all three species, males had relatively longer heads than females, while females were consistently heavier. However, the nature of dimorphism varied among species: in *A. tachyscopaeus*, differences were largely confined to head length and mass, whereas in *A. trachygaster*, dimorphism extended across a broader set of head dimensions, with females having wider and deeper heads than males. *Ailuronyx seychellensis* showed an intermediate pattern, with additional differences in toe length and eye-ear distance. The relatively longer heads of males across all species might be attributed to sexual selection due to greater bite force, hence enhancing success in male-male combat [92]. In contrast, the consistently greater mass of females might reflect selection based on fertility, as heavier females are likely to produce larger eggs [93]. Of the three species, *A. trachygaster* exhibited the most pronounced sexual dimorphism, which may be related to differences in social structure or ecological specialisation associated with its likely palm-specialist lifestyle [44]. Overall, the modest magnitude of dimorphism observed in *Ailuronyx* is consistent with patterns reported for other geckos [91], and the low explanatory power of sex in our models indicates that contribution of other factors to morphological variation is larger within this genus. However, further studies are needed to determine whether the observed species-specific patterns of dimorphism reflect differences in mating behaviour, territoriality, food niche partitioning or whether they are caused by other, not yet identified factors.

In *Ailuronyx*, species identification in the field has proven difficult, with frequent misidentification complicating assessments of their distribution and conservation status [10, 13, 30, 94, 95]. Our study extends the preliminary findings by Rocha et al. [13] by identifying multivariate morphometric traits that distinguish between currently described species of *Ailuronyx* geckos, allowing for reliable identification. To support this, we summarised differences in morphometric traits measurable in the field in Table S6. These differences may also facilitate visual identification of the currently recognised species in the field. However, because subtle traits can be difficult to assess visually, extensive observer training is essential to minimize misidentifications. Our results also suggest that some museum specimens might be misidentified, because we found discrepancies between our analysis and the assigned species in two of nine cases (Table S2). Based on our available dataset, discriminant analyses could be used to assess potential misidentification of other museum specimens. The µCT scan of the paratype of *A. tachyscopaeus* (BMNH 1907.10.15.54) revealed the absence of hemibacula, and external examination further showed no visible femoral pores and hemipenal bulges, indicating that this specimen is female rather than male, contrary to the original type description [43]. Furthermore, our work updates species distribution records, by reporting *A. tachyscopaeus* on two previously undocumented islands (Sainte Anne and Thérèse). Whether these records represent previous misidentifications or true new records is unclear. Although we found both species, *A. seychellensis* and *A. tachayscopaeus* on Thérèse, we only found the latter species to be present on Sainte Anne in our extended sampling. Similarly, the non-detection of *A. seychellensis* on many islands with historical records in more recent studies [13; this study], suggests the possibility of historical misidentifications or local extinctions. Despite intensive searches, no *Ailuronyx* could be detected on three of the visited islands (Marianne, Moyenne and Petite Soeur). While there are no previous records from Moyenne and Petite Soeur, on Marianne, historical records report the presence of *A. seychellensis* [27, and references therein]. Whether this species, or its possible misidentified sister species, is still present or has been lost on Marianne needs further investigation.

Conservation management relies on discrete categories, with species counts often considered as the measure to quantify biodiversity [96]. However, in the presence of cryptic diversity with variable knowledge of phenotypic, ecological and genetic diversity, defining conservation units can especially benefit the protection of isolated populations [8, 97]. While the three recognised *Ailuronyx* species are morphologically distinguishable, the substantial island-level variation within *A. seychellensis* and *A. tachyscopaeus*, and the pronounced divergence of the Mahé-south population of *A. tachyscopaeus*, suggest that conservation strategies focused solely on named species would overlook biologically meaningful diversity. We therefore suggest considering treating the identified northern and southern genetic clusters [13] as separate conservation units until more detailed genomic assessments provide further clarification. Additionally, we advise implementing conservation actions for the distinct Mahé-south population of *A. tachyscopaeus*, a unit which currently does not benefit from any conservation measures, and which is likely to be a very small, vulnerable population. These recommendations highlight the value of combining morphological, genetic, and ecological data when establishing conservation priorities in archipelagic systems, where cryptic diversity is prevalent.

## Conclusion

Our study has revealed notable morphometric divergences among Seychelles *Ailuronyx* geckos. This information is relevant for field-based surveys, where accurate species identification is essential for monitoring biodiversity and detecting range shifts. Incorporating our morphometric data into conservation assessments can therefore improve species monitoring by reducing species misidentification, and helping to prioritise habitat protection for morphologically, and possibly ecologically, distinct populations. Additionally, we uncovered substantial morphological variation at the island-level within both *A. seychellensis* and *A. tachyscopaeus*, as well as strong evidence for population-level divergence within *A. tachyscopaeus* on Mahé. Therefore, our findings contribute to understanding the evolutionary trajectories and adaptive processes of these endemic island lineages. Overall, our findings demonstrate how integrative morphometric approaches can uncover biologically meaningful variation among cryptic island lineages and emphasises the importance of island-scale perspectives when interpreting the evolutionary and biogeographic dynamics of archipelagic biodiversity.

## Supplementary Information

Below is the link to the electronic supplementary material.

Supplementary Material 1

## Data Availability

The specimens used in this study are curated in the collections specified in the Methods section and in Table S2. The µCT data generated and analysed in this study are available from LIB (https://media.leibniz-lib.de/) with the specimen specific DOIs listed in Table S2, and MorphoSource (https://www.morphosource.org/projects/000804041). All additional data and code used for the analysis are deposited on Zenodo (10.5281/zenodo.17361415).

## Abbreviations

2D: Two-dimensional
3D: Three-dimensional
µCT: micro-computed tomography
CS: Centroid size
PCA: Principal Component Analysis
CREA: Craniofacial evolutionary allometry
