# Supplementary material for: Inter- and intraspecific morphometric divergences in a Seychelles endemic gecko genus
Source: BMC Ecol Evol. 2026 Apr 21;26:38. doi: 10.1186/s12862-026-02517-9 (PMC13097663; doi:10.1186/s12862-026-02517-9)
Supplement: Supplementary file 1 — Supplementary Material 1 [file 12862_2026_2517_MOESM1_ESM.docx]

**SUPPLEMENTARY MATERIALS**

**Inter- and intraspecific morphometric divergences in a Seychelles endemic gecko genus**

Markus A. Roesch^1,2,3,4*^, Nancy Bunbury^4,5^, D. James Harris^1,3^, Sara Rocha^6^, Christopher N. Kaiser-Bunbury^5^, David J. Gower^7,8^, Greg Berke^9^, Christina Marques^10^, Gérard Rocamora^8^, Anna Zora^11^, & Karolin Engelkes^12^

*Corresponding author. E-Mail: markus.roesch@cibio.up.pt

| **Table S1.** Summary of field sampling effort across the 18 surveyed islands. | | | | | | | |
| --- | --- | --- | --- | --- | --- | --- | --- |
| **Island** | **Island Group** | **Island size (ha)*** | **Sampling period** | **Number of visits** | **Search effort (person-days)**** | **Number of collectors** | **Number of geckos sampled** |
| Aride | Northern granitic | 73 | February 2024, July–August 2024 | 2 | 28 | 1–2 | 50 |
| Cousine | Northern granitic | 26 | March 2024, August–September 2024 | 2 | 14 | 1 | 53 |
| Curieuse | Northern granitic | 286 | April–May 2024 | 1 | 7 | 1–2 | 19 |
| Félicité | Northern granitic | 230 | April 2024 | 1 | 7 | 1 | 25 |
| Frégate | Northern granitic | 219 | January–February 2024, August 2024 | 2 | 30 | 1–2 | 55 |
| Grande Soeur | Northern granitic | 85 | March–April 2024 | 1 | 4 | 1 | 21 |
| La Digue | Northern granitic | 1,000 | April 2024, October 2024, Decembre 2024 | 3 | 22 | 1–2 | 17 |
| Marianne | Northern granitic | 100 | March 2024 | 1 | 12 | 4 | 0 |
| Petite Soeur | Northern granitic | 35 | March 2024 | 1 | 12 | 4 | 0 |
| Praslin | Northern granitic | 3,850 | January 2024 – January 2025 | Resident | 100+ | 1–4 | 233 |
| Anonyme | Southern granitic | 10 | Decembre 2024 | 1 | 3 | 1 | 15 |
| Cérf | Southern granitic | 131 | Decembre 2024 | 1 | 1 | 1 | 8 |
| Conception | Southern granitic | 69 | October 2024 | 1 | 2 | 2 | 5 |
| Mahé | Southern granitic | 15,252 | May 2024, September–October 2024, January 2025 | 3 | 36 | 1–2 | 38 |
| Moyenne | Southern granitic | 9 | November 2024 | 1 | 4 | 2 | 0 |
| Sainte Anne | Southern granitic | 219 | May 2024 | 1 | 4 | 2 | 14 |
| Silhouette | Southern granitic | 1,995 | February–March 2024, July 2024 | 2 | 38 | 1–2 | 57 |
| Therese | Southern granitic | 74 | October 2024 | 1 | 2 | 2 | 8 |
| * Rocamora G. and Henriette E. Invasive Alien Species in Seychelles. Why and how to eliminate them? Identification and management of priority species. Muséum national d'Histoire naturelle, Paris, Biotope, Mèze, 384 p., 2015.  ** We define person-day as ~10 hours of work. | | | | | | | |

| **Table S2.** List of material used in the geometric morphometric analyses with information on the micro-computed tomography parameters used to scan the specimens. Asterisk (*) indicates that these specimens were identified as *A. seychellensis* in the ZFMK collection, but our evaluation and analysis consistently clustered them within *A. tachyscopaeus*. Catalogue abbreviations: BMNH (Natural History Museum of London), CAS (California Academy of Sciences) and ZFMK (Zoological Research Museum Alexander Koenig). | | | | | | | | | | |
| --- | --- | --- | --- | --- | --- | --- | --- | --- | --- | --- |
| Species | Catalogue Number | Field Number | Island | Voxel (µm) | Voltage (kV) | Current (μA) | Filter | Exp.Time (msecs) | CT Scanner | DOI/ARK |
| *A. seychellensis* | CAS:HERP:167549 | *NA* | Aride | 30.630 | 70 | *NA* | *NA* | *NA* | Zeiss Xradia microXCT-400 | ark:/87602/m4/M101124 |
| *A. seychellensis* | BMNH 1976.1864 | *NA* | Cousin | 27.098 | *NA* | *NA* | *NA* | *NA* | Nikon Metrology HMX ST 225 microCT | ark:/87602/m4/804204 |
| *A. seychellensis* | BMNH 1976.1866 | *NA* | Cousin | 26.117 | *NA* | *NA* | *NA* | *NA* | Nikon Metrology HMX ST 225 microCT | ark:/87602/m4/804491 |
| *A. seychellensis* | ZFMK-HERP-42795 | *NA* | Praslin | 10.218 | 150 | 35 | 0.2mm Cu | 111.11 | YXLON FF20 CT | https://doi.org/10.20363/Ailuronyx2025-ct-h-ZFMK-HERP-42795 |
| *A. seychellensis* | ZFMK-HERP-43824 | *NA* | Mahé | 9.557 | 150 | 35 | 0.2mm Cu | 111.11 | YXLON FF20 CT | https://doi.org/10.20363/Ailuronyx2025-ct-h-ZFMK-HERP-43824 |
| *A. seychellensis* | ZFMK-HERP-45018 | *NA* | Mahé | 12.162 | 150 | 35 | 0.2mm Cu | 111.11 | YXLON FF20 CT | https://doi.org/10.20363/Ailuronyx2025-ct-h-ZFMK-HERP-45018 |
| *A. seychellensis* | ZFMK-HERP-45782 | *NA* | Cousin | 12.296 | 150 | 35 | 0.2mm Cu | 111.11 | YXLON FF20 CT | https://doi.org/10.20363/Ailuronyx2025-ct-h-ZFMK-HERP-45782 |
| *A. seychellensis* | ZFMK-HERP-45783 | *NA* | Praslin | 12.990 | 150 | 35 | 0.2mm Cu | 111.11 | YXLON FF20 CT | https://doi.org/10.20363/Ailuronyx2025-ct-h-ZFMK-HERP-45783 |
| *A. seychellensis* | ZFMK-HERP-45784 | *NA* | Praslin | 9.122 | 150 | 35 | 0.2mm Cu | 111.11 | YXLON FF20 CT | https://doi.org/10.20363/Ailuronyx2025-ct-h-ZFMK-HERP-45784 |
| *A. seychellensis* | ZFMK-HERP-104218 | AN.SEY.15 | Anonyme | 10.564 | 160 | 50 | 0.2mm Cu | 111.11 | YXLON FF20 CT | https://doi.org/10.20363/Ailuronyx2025-ct-h-ZFMK-HERP-104218 |
| *A. seychellensis* | ZFMK-HERP-104219 | AR.SEY.01 | Aride | 14.769 | 150 | 35 | 0.2mm Cu | 111.11 | YXLON FF20 CT | https://doi.org/10.20363/Ailuronyx2025-ct-h-ZFMK-HERP-104219 |
| *A. seychellensis* | ZFMK-HERP-104222 | CE.SEY.01 | Cousine | 14.769 | 150 | 35 | 0.2mm Cu | 111.11 | YXLON FF20 CT | https://doi.org/10.20363/Ailuronyx2025-ct-h-ZFMK-HERP-104222 |
| *A. seychellensis* | ZFMK-HERP-104225 | FR.SEY.01 | Frégate | 12.163 | 160 | 50 | 0.2mm Cu | 111.11 | YXLON FF20 CT | https://doi.org/10.20363/Ailuronyx2025-ct-h-ZFMK-HERP-104225 |
| *A. seychellensis* | ZFMK-HERP-104228 | MA.SEY.04 | Mahe | 11.025 | 160 | 50 | 0.2mm Cu | 111.11 | YXLON FF20 CT | https://doi.org/10.20363/Ailuronyx2025-ct-h-ZFMK-HERP-104228 |
| *A. seychellensis* | ZFMK-HERP-104231 | PR.SEY.32 | Praslin | 12.597 | 150 | 35 | 0.2mm Cu | 111.11 | YXLON FF20 CT | https://doi.org/10.20363/Ailuronyx2025-ct-h-ZFMK-HERP-104231 |
| *A. seychellensis* | ZFMK-HERP-104236 | SI.SEY.01 | Silhouette | 12.597 | 150 | 35 | 0.2mm Cu | 111.11 | YXLON FF20 CT | https://doi.org/10.20363/Ailuronyx2025-ct-h-ZFMK-HERP-104236 |
| *A. seychellensis* | ZFMK-HERP-104239 | TH.SEY.02 | Thérèse | 10.008 | 150 | 50 | 0.2mm Cu | 111.11 | YXLON FF20 CT | https://doi.org/10.20363/Ailuronyx2025-ct-h-ZFMK-HERP-104239 |
| *A. tachyscopaeus* | BMNH 1907.10.15.54 | *NA* | Praslin | 17.641 | *NA* | *NA* | *NA* | *NA* | Nikon Metrology HMX ST 225 microCT | ark:/87602/m4/806128 |
| *A. tachyscopaeus* | ZFMK-HERP-33426* | *NA* | Praslin | 9.556 | 145 | 35 | 0.2mm Cu | 111.11 | YXLON FF20 CT | https://doi.org/10.20363/Ailuronyx2025-ct-h-ZFMK-HERP-33426 |
| *A. tachyscopaeus* | ZFMK-HERP-46726* | *NA* | Praslin | 9.556 | 150 | 35 | 0.2mm Cu | 111.11 | YXLON FF20 CT | https://doi.org/10.20363/Ailuronyx2025-ct-h-ZFMK-HERP-46726 |
| *A. tachyscopaeus* | ZFMK-HERP-104220 | CR.TAC.04 | Cérf | 8.274 | 145 | 35 | 0.2mm Cu | 111.11 | YXLON FF20 CT | https://doi.org/10.20363/Ailuronyx2025-ct-h-ZFMK-HERP-104220 |
| *A. tachyscopaeus* | ZFMK-HERP-104221 | CO.TAC.02 | Conception | 8.253 | 140 | 35 | 0.2mm Cu | 111.11 | YXLON FF20 CT | https://doi.org/10.20363/Ailuronyx2025-ct-h-ZFMK-HERP-104221 |
| *A. tachyscopaeus* | ZFMK-HERP-104223 | CU.TAC.08 | Curieuse | 12.163 | 140 | 50 | 0.3mm Cu | 111.11 | YXLON FF20 CT | https://doi.org/10.20363/Ailuronyx2025-ct-h-ZFMK-HERP-104223 |
| *A. tachyscopaeus* | ZFMK-HERP-104224 | FE.TAC.01 | Félicité | 10.220 | 90 | 80 | - | 111.11 | YXLON FF20 CT | https://doi.org/10.20363/Ailuronyx2025-ct-h-ZFMK-HERP-104224 |
| *A. tachyscopaeus* | ZFMK-HERP-104226 | GS.TAC.01 | Grande Soeur | 9.134 | 145 | 35 | 0.2mm Cu | 111.11 | YXLON FF20 CT | https://doi.org/10.20363/Ailuronyx2025-ct-h-ZFMK-HERP-104226 |
| *A. tachyscopaeus* | ZFMK-HERP-104227 | LD.TAC.11 | La Digue | 10.056 | 120 | 60 | 0.1mm CU | 111.11 | YXLON FF20 CT | https://doi.org/10.20363/Ailuronyx2025-ct-h-ZFMK-HERP-104227 |
| *A. tachyscopaeus* | ZFMK-HERP-104229 | MA.TAC.14 | Mahé (north) | 8.688 | 150 | 35 | 0.2mm Cu | 111.11 | YXLON FF20 CT | https://doi.org/10.20363/Ailuronyx2025-ct-h-ZFMK-HERP-104229 |
| *A. tachyscopaeus* | ZFMK-HERP-104230 | MA.TAC.11 | Mahé (south) | 9.556 | 140 | 30 | 0.2mm Cu | 111.11 | YXLON FF20 CT | https://doi.org/10.20363/Ailuronyx2025-ct-h-ZFMK-HERP-104230 |
| *A. tachyscopaeus* | ZFMK-HERP-104232 | PR.TAC.32 | Praslin | 11.173 | 140 | 45 | 0.2mm Cu | 111.11 | YXLON FF20 CT | https://doi.org/10.20363/Ailuronyx2025-ct-h-ZFMK-HERP-104232 |
| *A. tachyscopaeus* | ZFMK-HERP-104235 | SA.TAC.11 | Sainte Anne | 9.099 | 160 | 35 | none | 111.11 | YXLON FF20 CT | https://doi.org/10.20363/Ailuronyx2025-ct-h-ZFMK-HERP-104235 |
| *A. tachyscopaeus* | ZFMK-HERP-104237 | SI.TAC.04 | Silhouette | 9.991 | 145 | 35 | 0.2mm Cu | 111.11 | YXLON FF20 CT | https://doi.org/10.20363/Ailuronyx2025-ct-h-ZFMK-HERP-104237 |
| *A. tachyscopaeus* | ZFMK-HERP-104238 | SI.TAC.01 | Silhouette | 9.511 | 145 | 35 | 0.2mm Cu | 111.11 | YXLON FF20 CT | https://doi.org/10.20363/Ailuronyx2025-ct-h-ZFMK-HERP-104238 |
| *A. tachyscopaeus* | ZFMK-HERP-104240 | TH.TAC.01 | Thérèse | 9.556 | 140 | 30 | 0.2mm Cu | 111.11 | YXLON FF20 CT | https://doi.org/10.20363/Ailuronyx2025-ct-h-ZFMK-HERP-104240 |
| *A. trachygaster* | ZFMK-HERP-104233 | LH-TRA-01 | Praslin | 25.329 | 160 | 50 | 0.2mm Cu | 111.11 | YXLON FF20 CT | https://doi.org/10.20363/Ailuronyx2025-ct-h-ZFMK-HERP-104233 |
| *A. trachygaster* | ZFMK-HERP-104234 | LH-TRA-02 | Praslin | 18.244 | 150 | 35 | 0.2mm Cu | 111.11 | YXLON FF20 CT | https://doi.org/10.20363/Ailuronyx2025-ct-h-ZFMK-HERP-104234 |
| *A. trachygaster* | ZFMK-HERP-104241 | LH-TRA-03 | Praslin | 18.534 | 150 | 35 | 0.2mm Cu | 111.11 | YXLON FF20 CT | https://doi.org/10.20363/Ailuronyx2025-ct-h-ZFMK-HERP-104241 |
| *A. trachygaster* | ZFMK-HERP-104242 | LH-TRA-04 | Praslin | 13.901 | 150 | 35 | 0.2mm Cu | 111.11 | YXLON FF20 CT | https://doi.org/10.20363/Ailuronyx2025-ct-h-ZFMK-HERP-104242 |
| *A. trachygaster* | *NA* | HK-TRA-01 | Praslin | 22.153 | 150 | 35 | 0.2mm Cu | 111.11 | YXLON FF20 CT | ark:/87602/m4/806134 |
| *A. trachygaster* | *NA* | HK-TRA-02 | Praslin | 33.882 | 150 | 35 | 0.2mm Cu | 111.11 | YXLON FF20 CT | ark:/87602/m4/806239 |
| *A. trachygaster* | *NA* | HK-TRA-03 | Praslin | 20.198 | 150 | 35 | 0.2mm Cu | 111.11 | YXLON FF20 CT | ark:/87602/m4/806245 |
| *A. trachygaster* | *NA* | HK-TRA-04 | Praslin | 20.850 | 150 | 35 | 0.2mm Cu | 111.11 | YXLON FF20 CT | ark:/87602/m4/806984 |
| *A. trachygaster* | *NA* | HK-TRA-05 | Praslin | 21.284 | 150 | 35 | 0.2mm Cu | 111.11 | YXLON FF20 CT | ark:/87602/m4/806260 |

| **Table S3.** Description of the 3D landmarks used in this study. M= represent mid-sagittal plane landmarks. | | |
| --- | --- | --- |
| **ID** | **Homologue** | **Description** |
| **Cranium** |  |  |
| 1 | M | Anteriormost point of premaxilla |
| 2 | 67 | Anterior, lower edge of premaxilla on maxillary facet |
| 3 | 68 | Anterior, upper edge of premaxilla on maxillary facet |
| 4 | 69 | Anteriormost edge of the premaxillary-nasal suture |
| 5 | M | Center of the posteriormost point of the premaxilla, on the ascendent process |
| 6 | 70 | Anterolateralmost point of the nasal, on the maxillary-nasal suture |
| 7 | 71 | Anterodorsalmost point of the maxillary, on the maxilla spur |
| 8 | 72 | Inner boarder of anteriormost supralabial foramina |
| 9 | 73 | Anteriorlateralmost point of the frontal, in the frontal-nasal-prefrontal suture |
| 10 | 74 | Posteriordorsalmost point of the maxilla, in the maxilla-frontal-prefrontal suture |
| 11 | M | Center of frontal-nasal suture on anteromedial process of the frontal |
| 12 | 75 | Dorsalmost point of the prefrontal, on dorsal process |
| 13 | 76 | Dorsalmost point of maxilla to the posteroventral process of the prefrontal |
| 14 | 77 | Posteriormost point of the prefrontal on posteroventral process |
| 15 | 78 | Posteriormost point of the maxilla, on posterior process |
| 16 | 79 | Posteriormost point of the jugal |
| 17 | M | Center of frontoparietal notch, in the frontal suture |
| 18 | 80 | Anteriordorsalmost edge of postorbitofrontal in the postorbitofrontal-frontal suture |
| 19 | 81 | Anteriorlaeralmost point of parietal at parietal-frontal-postorbitofrontal suture |
| 20 | 82 | Ventralmost point of the postorbitofrontal on postfrontal spur |
| 21 | 83 | Dorsalmost point of postorbitofrontal to the postparietal process |
| 22 | 84 | Posteriormost point of postorbitofrontal on the postfrontal posterior process |
| 23 | 85 | Lateralmost point of parietal on the postfrontal posterior process |
| 24 | 86 | Anteriormost point of squamosal |
| 25 | 87 | Medial point in insertion of the posterior process of the parietal |
| 26 | M | Center of parietal notch |
| 27 | M | Center of the dorsalmost point of the supraoccipital |
| 28 | 88 | Lateralmost point of the supraoccipital |
| 29 | M | Center of the dorsalmost point of the foramen magnum |
| 30 | 89 | Dorsolateralmost point of the posterior process of the parietal |
| 31 | 90 | Posteriormost point of squamosal |
| 32 | 91 | Posteriorlateralmost point of the paraoccipital process |
| 33 | 92 | Anterolateralmost point of the mandibular condyle of the quadrate |
| 34 | 93 | Posterolateralmost point of the mandibular condyle of the quadrate |
| 35 | 94 | Posteriormost point of the pterygoid |
| 36 | 95 | Posterior end of the sutural surface of the dorsal quadrate epiphysis |
| 37 | 96 | Medialmost point of the spheno-occipital tubercle |
| 38 | 97 | Dorsalmost point of the occipital condyle |
| 39 | M | Center of posteroventral edge of occipital condyle |
| 40 | M | Posteriormost point of the medial groove on the premaxilla |
| 41 | 98 | Ventral posteriormost point of premaxilla on palatal process |
| 42 | 99 | Ventral anteromedial most point of maxilla, on the maxillary lappet |
| 43 | 100 | Posteromedialmost point of the palatine process of vomer |
| 44 | 101 | Anteriormost point of the vomerine process of the palatine |
| 45 | 102 | posteriormost point of vomer on palatine facet |
| 46 | M | Anteriormedialmost point of the subolfatory canal on the frontal |
| 47 | 103 | Anterolateral edge of the palatine, on lateral process |
| 48 | 104 | Anteriormost point of the ectopterygoid |
| 49 | 105 | Lateralmost point of the septomaxilla at palatine notch |
| 50 | 106 | Anterolateral edge of the pterygoid, on the ectopterygoid process |
| 51 | 107 | Posterolateral edge of the ectopterygoid |
| 52 | 108 | Lateralmost point of the septomaxilla on the pterygoid shelf |
| 53 | 109 | Anterolateralmost point of the pterygoid on the ectopterygoid process |
| 54 | 110 | Medialmost point of the septomaxilla on the pterygoid shelf |
| 55 | 111 | Anterolateral edge of the pterygoid on the palatine process |
| 56 | M | Posteromedialmost point of the subolfatory canal on the frontal |
| 57 | M | Center of the anteriormost point of the parabasisphenoid, between the trabeculae |
| 58 | 112 | Anteriormost point of basipterygoid process |
| 59 | 113 | Posterolateralmost point of basipterygoid process |
| 60 | 114 | Posteriormost point of basipterygoid process |
| 61 | 115 | Lateralmost point of the crista prootica |
| 62 | 116 | Dorsalmost end of the VII facial branch foramen |
| 63 | 117 | Anteroventralmost point of the epipterygoid on the pterygoid process |
| 64 | 118 | Anteriormost point of the crista alaris |
| 65 | 119 | Lateral edge of the otostapes |
| 66 | 120 | Anteriormost point of the footplate of the otostapes |
| **Mandible** |  |  |
| 1 | 25 | Anterior (labial) surface of anteriormost point of dentary |
| 2 | 26 | Posterior (lingual) surface of anteriormost point of dentary |
| 3 | 27 | Dorsally to the posteriormost point of dentary, on the cornoid process |
| 4 | 28 | Anterolateralmost point of the coronoid, on the anterolateral process |
| 5 | 29 | Laterally the posteriormost point of dentary, on the superior process |
| 6 | 30 | Anteriormost end of the surangular foramen |
| 7 | 31 | Laterally to the anteriormost point of surangular |
| 8 | 32 | Ventrally to the posteriormost point of dentary, on the inferior process |
| 9 | 33 | Posteroventralmost point of splenial |
| 10 | 34 | Anteroventralmost point of splenial |
| 11 | 35 | Posterior end of the anterior inferior alveolar foramen |
| 12 | 36 | Posteroventralmost point of splenial at prearticular process of compound bone |
| 13 | 37 | Anteromedialmost point of the coronoid, on the anteromedial process |
| 14 | 38 | Ventralmost point of the lateral ridge of the coronoid process |
| 15 | 39 | Dorsalmost point of coronoid |
| 16 | 40 | Posteriormost point of coronoid |
| 17 | 41 | Anterior insertion of the mandibular fossa |
| 18 | 42 | Posterior end of the aperture of the mandibula fenestra |
| 19 | 43 | Posterior end of the surangular foramen |
| 20 | 44 | Lateralmost point of the articular surface |
| 21 | 45 | Dorsalmost point of the articular surface |
| 22 | 46 | Ventral end of the foramen for the chorda tympani |
| 23 | 47 | Posterolateralmost point of the retroarticular process |
| 24 | 48 | Posteromedialmost point of the retroarticular process |

| **Table S4.** Results from the sensitivity analysis of permutational multivariate tests to explore the effect of species, sex and geographical region on shape variation of the 2D datasets including sexed and unsexed, adult and presumed juvenile individuals. Vars = Matrix of response variables (i.e. size-corrected morphometric measurements) included in the model (see description of variables in the material and methods in the main text). Sex = Female, male and unsexed, Region = northern and southern granitic islands, and north-south Mahé.  Significant results are in bold. | | | | | | | | |
| --- | --- | --- | --- | --- | --- | --- | --- | --- |
| Model | Variable | Df | SS | MS | *R^2^* | F | Z | *P value* |
| **2D morphometrics including presumed juveniles** | | |  |  |  |  |  |  |
| Vars ~ Species * Sex + Island | Species | 2 | 4421.3 | 2210.65 | 0.71774 | 1174.9957 | 11.9562 | **< 0.001** |
|  | Sex | 2 | 334.9 | 167.45 | 0.05437 | 89.0001 | 10.1948 | **< 0.001** |
|  | Island | 15 | 224.4 | 14.96 | 0.03643 | 7.9509 | 12.8852 | **< 0.001** |
|  | Species:Sex | 4 | 63.7 | 15.93 | 0.01035 | 8.4692 | 8.2402 | **< 0.001** |
|  | Residuals | 593 | 1115.7 | 1.88 | 0.18112 |  |  |  |
|  | Total | 616 | 6160.0 |  |  |  |  |  |
| *A. seychellensis* | |  |  |  |  |  |  |  |
| Vars ~ Sex + Region/Island | Sex | 2 | 310.66 | 155.328 | 0.12328 | 22.7577 | 7.7187 | **< 0.001** |
|  | Region | 1 | 60.92 | 60.919 | 0.02417 | 0.7461 | -0.3205 | 0.6235 |
|  | Region:Island | 6 | 489.8 | 81.647 | 0.19440 | 11.9624 | 12.3146 | **< 0.001** |
|  | Residuals | 243 | 1658.54 | 6.825 | 0.65815 |  |  |  |
|  | Total | 252 | 2520.00 |  |  |  |  |  |
| *A. tachyscopaeus* | |  |  |  |  |  |  |  |
| Vars ~ Sex + Region/Island | Sex | 2 | 296.81 | 148.406 | 0.13370 | 20.9026 | 8.6130 | **< 0.001** |
|  | Region | 1 | 44.23 | 44.232 | 0.01992 | 1.1196 | 0.3373 | 0.3654 |
|  | Region:Island | 10 | 395.08 | 39.508 | 0.17796 | 5.5646 | 9.2936 | **< 0.001** |
|  | Residuals | 209 | 1483.87 | 7.100 | 0.66841 |  |  |  |
|  | Total | 222 | 2220.00 |  |  |  |  |  |
| *A. tachyscopaeus* Mahé | |  |  |  |  |  |  |  |
| Vars ~ Sex + Region | Sex | 2 | 65.864 | 32.932 | 0.34476 | 8.2217 | 3.3909 | **< 0.001** |
|  | Region | 1 | 41.062 | 41.062 | 0.21494 | 10.2513 | 3.2698 | **< 0.001** |
|  | Residuals | 21 | 84.116 | 4.006 | 0.44030 |  |  |  |
|  | Total | 24 | 191.042 |  |  |  |  |  |
| *A. trachygaster* |  |  |  |  |  |  |  |  |
| Vars ~ Sex | Sex | 2 | 469.35 | 234.676 | 0.33525 | 34.799 | 7.8106 | **< 0.001** |
|  | Residuals | 138 | 930.65 | 6.744 | 0.66475 |  |  |  |
|  | Total | 140 | 1400.00 |  |  |  |  |  |

| **Table S5.** Results from permutational multivariate tests to explore the effect of size (centroid size: CS), species, sex and geographical region on shape variation of the different cranial, mandibular and 2D datasets. Shape = Symmetric component of 3D landmarks sets after general Procrustes analysis. Vars = Matrix of response variables (i.e. size-corrected morphometric measurements) included in the model (see description of variables in the material and methods in the main text). Sex = Female and male, Region = northern and southern granitic islands, and north-south Mahé.  Significant results are in bold. | | | | | | | | |
| --- | --- | --- | --- | --- | --- | --- | --- | --- |
| Model | Variable | Df | SS | MS | *R^2^* | F | Z | *P value* |
| **3D geometric morphometrics** | |  |  |  |  |  |  |  |
| **Cranium** |  |  |  |  |  |  |  |  |
| Shape ~ log(CS) + Species * Sex + Island | log(CS) | 1 | 0.0819 | 0.0819 | 0.5224 | 67.6132 | 3.8145 | **< 0.001** |
|  | Species | 2 | 0.0282 | 0.0141 | 0.1797 | 11.6284 | 5.2459 | **< 0.001** |
|  | Sex | 1 | 0.0026 | 0.0026 | 0.0165 | 2.1337 | 1.8732 | **0.0312** |
|  | Island | 16 | 0.0165 | 0.0010 | 0.1050 | 0.8494 | -1.1253 | 0.8678 |
|  | Species:Sex | 2 | 0.0046 | 0.0023 | 0.0296 | 1.9136 | 1.7384 | **0.0413** |
|  | Residuals | 19 | 0.0230 | 0.0012 | 0.1468 |  |  |  |
|  | Total | 41 | 0.1569 |  |  |  |  |  |
| *A. seychellensis* | |  |  |  |  |  |  |  |
| Shape ~ log(CS) + Sex + Region | log(CS) | 1 | 0.0038 | 0.0038 | 0.1331 | 2.7250 | 2.0983 | **0.0181** |
|  | Sex | 1 | 0.0049 | 0.0049 | 0.1723 | 3.5281 | 2.3749 | **0.0072** |
|  | Region | 1 | 0.0017 | 0.0017 | 0.0599 | 1.2271 | 0.6875 | 0.2425 |
|  | Residuals | 13 | 0.0179 | 0.0014 | 0.6348 |  |  |  |
|  | Total | 16 | 0.0282 |  |  |  |  |  |
| *A. tachyscopaeus* | |  |  |  |  |  |  |  |
| Shape ~ log(CS) + Sex + Region | log(CS) | 1 | 0.0024 | 0.0024 | 0.0024 | 3.2823 | 2.9495 | **0.0002** |
|  | Sex | 1 | 0.0009 | 0.0009 | 0.0657 | 1.2056 | 0.7012 | 0.2108 |
|  | Region | 1 | 0.0013 | 0.0013 | 0.1009 | 1.8499 | 1.6061 | 0.0598 |
|  | Residuals | 12 | 0.0086 | 0.0007 | 0.6544 |  |  |  |
|  | Total | 15 | 0.0132 |  |  |  |  |  |
| **Mandible** |  |  |  |  |  |  |  |  |
| Shape ~ log(CS) + Species * Sex + Island | log(CS) | 1 | 0.1502 | 0.1502 | 0.6628 | 120.2737 | 4.0480 | **< 0.001** |
|  | Species | 2 | 0.0309 | 0.0154 | 0.1364 | 12.3709 | 4.5637 | **< 0.001** |
|  | Sex | 1 | 0.0010 | 0.0010 | 0.0044 | 0.7974 | -0.0988 | 0.5332 |
|  | Island | 16 | 0.0185 | 0.0012 | 0.0815 | 0.9237 | -0.3959 | 0.6508 |
|  | Species:Sex | 2 | 0.0023 | 0.0012 | 0.0103 | 0.9302 | 0.1053 | 0.4549 |
|  | Residuals | 19 | 0.0237 | 0.0012 | 0.1047 |  |  |  |
|  | Total | 41 | 0.2266 |  |  |  |  |  |
| *A. seychellensis* | |  |  |  |  |  |  |  |
| Shape ~ log(CS) + Sex + Region | log(CS) | 1 | 0.0030 | 0.0030 | 0.1195 | 2.1000 | 1.6185 | 0.0547 |
|  | Sex | 1 | 0.0021 | 0.0021 | 0.0839 | 1.4746 | 0.9265 | 0.1833 |
|  | Region | 1 | 0.0014 | 0.0014 | 0.0568 | 0.9985 | 0.2926 | 0.3838 |
|  | Residuals | 13 | 0.0188 | 0.0014 | 0.7398 |  |  |  |
|  | Total | 16 | 0.0254 |  |  |  |  |  |
| *A. tachyscopaeus* | |  |  |  |  |  |  |  |
| Shape ~ log(CS) + Sex + Region | log(CS) | 1 | 0.0029 | 0.0029 | 0.1935 | 3.5274 | 2.7400 | **0.0016** |
|  | Sex | 1 | 0.0006 | 0.0006 | 0.0424 | 0.7726 | -0.3017 | 0.6079 |
|  | Region | 1 | 0.0016 | 0.0016 | 0.1061 | 1.9348 | 1.4854 | 0.0741 |
|  | Residuals | 12 | 0.0098 | 0.0008 | 0.6581 |  |  |  |
|  | Total | 15 | 0.0149 |  |  |  |  |  |
| **2D morphometrics** | | |  |  |  |  |  |  |
| Vars ~ Species * Sex + Island | Species | 2 | 4343.4000 | 2171.7100 | 0.7674 | 1182.2866 | 11.7334 | **< 0.001** |
|  | Sex | 1 | 56.9000 | 56.8900 | 0.0101 | 30.9687 | 6.2193 | **< 0.001** |
|  | Island | 15 | 236.7000 | 15.7800 | 0.0418 | 8.5894 | 14.2695 | **< 0.001** |
|  | Species:Sex | 2 | 20.1000 | 10.0500 | 0.0036 | 5.4686 | 4.4956 | **< 0.001** |
|  | Residuals | 546 | 1002.9000 | 1.8400 | 0.1772 |  |  |  |
|  | Total | 566 | 5660.0000 |  |  |  |  |  |
| *A. seychellensis* | |  |  |  |  |  |  |  |
| Vars ~ Sex + Region/Island | Sex | 1 | 87.2500 | 87.2500 | 0.0356 | 12.4046 | 5.5456 | **< 0.001** |
|  | Region | 1 | 67.6500 | 67.6520 | 0.0276 | 0.6462 | -0.5512 | 0.7096 |
|  | Region:Island | 6 | 628.1100 | 104.6840 | 0.2564 | 14.8832 | 13.0758 | **< 0.001** |
|  | Residuals | 237 | 1666.9900 | 7.0340 | 0.6804 |  |  |  |
|  | Total | 245 | 2450.0000 |  |  |  |  |  |
| *A. tachyscopaeus* | |  |  |  |  |  |  |  |
| Vars ~ Sex + Region/Island | Sex | 1 | 63.3900 | 63.3940 | 0.0309 | 8.4072 | 4.4552 | **< 0.001** |
|  | Region | 1 | 51.0300 | 51.0320 | 0.0249 | 1.0626 | 0.2808 | 0.3875 |
|  | Region:Island | 10 | 480.2600 | 48.0260 | 0.2343 | 6.3692 | 10.2101 | **< 0.001** |
|  | Residuals | 193 | 1455.3100 | 7.5400 | 0.7099 |  |  |  |
|  | Total | 205 | 2050.0000 |  |  |  |  |  |
| *A. tachyscopaeus* Mahé | |  |  |  |  |  |  |  |
| Vars ~ Sex + Region | Sex | 1 | 15.6990 | 15.6990 | 0.1085 | 3.4638 | 1.9976 | **0.0237** |
|  | Region | 1 | 38.3500 | 38.3500 | 0.2650 | 8.4615 | 3.2173 | **< 0.001** |
|  | Residuals | 20 | 90.6470 | 4.5320 | 0.6265 |  |  |  |
|  | Total | 22 | 144.6960 |  |  |  |  |  |
| *A. trachygaster* |  |  |  |  |  |  |  |  |
| Vars ~ Sex | Sex | 1 | 90.7000 | 90.7020 | 0.0796 | 9.7678 | 5.8160 | **< 0.001** |
|  | Residuals | 113 | 1049.3000 | 9.2860 | 0.9204 |  |  |  |
|  | Total | 114 | 1140.0000 |  |  |  |  |  |

| **Table S6.** Summary of2D morphometric measurements of the currently recognised *Ailuronyx* species in Seychelles. N = number of measured geckos. Values represent raw data before body size-correction and are given as mean ± SD. | | | | | | |
| --- | --- | --- | --- | --- | --- | --- |
|  | *Ailuronyx seychellensis* | | *Ailuronyx tachyscopaeus* | | *Ailuronyx trachygaster* | |
|  | Female | Male | Female | Male | Female | Male |
| N | 116 | 130 | 100 | 106 | 69 | 46 |
| Eye diameter (mm) | 6.68 ± 0.54 | 6.74 ± 0.55 | 4.83 ± 0.49 | 4.75 ± 0.49 | 8.17 ± 0.50 | 7.90 ± 0.54 |
| Eye-ear distance (mm) | 6.74 ± 0.68 | 6.92 ± 0.74 | 4.60 ± 0.51 | 4.42 ± 0.43 | 12.05 ± 0.69 | 11.19 ± 0.69 |
| Fourth toe length (mm) | 12.72 ± 1.46 | 12.83 ± 1.31 | 8.38 ± 0.66 | 8.00 ± 0.64 | 21.77 ± 1.47 | 20.55 ± 1.41 |
| Head height (mm) | 11.18 ± 0.78 | 11.26 ± 0.86 | 7.51 ± 0.56 | 7.23 ± 0.53 | 16.20 ± 0.86 | 15.35 ± 0.75 |
| Head length (mm) | 24.62 ± 1.59 | 24.67 ± 1.68 | 18.17 ± 1.29 | 17.65 ± 1.16 | 36.17 ± 1.39 | 34.32 ± 1.17 |
| Head width (mm) | 17.45 ± 1.34 | 17.49 ± 1.44 | 11.77 ± 0.91 | 11.40 ± 0.89 | 30.50 ± 1.40 | 28.77 ± 1.32 |
| Internarial distance (mm) | 2.74 ± 0.23 | 2.78 ± 0.27 | 1.91 ± 0.22 | 1.85 ± 0.20 | 4.99 ± 0.31 | 4.75 ± 0.37 |
| Interorbital distance (mm) | 5.81 ± 0.93 | 6.01 ± 0.98 | 3.40 ± 0.53 | 3.39 ± 0.61 | 11.31 ± 1.16 | 10.61 ± 0.89 |
| Snout-eye distance (mm) | 11.39 ± 0.81 | 11.44 ± 0.85 | 8.66 ± 0.62 | 8.36 ± 0.66 | 16.45 ± 0.68 | 15.55 ± 0.54 |
| Snout-vent length (mm) | 110.91 ± 8.40 | 109.40 ± 8.94 | 76.19 ± 5.67 | 72.32 ± 4.93 | 158.59 ± 6.94 | 147.63 ± 4.86 |
| Weight (g) | 38.78 ± 8.95 | 37.92 ± 9.85 | 11.21 ± 2.66 | 9.31 ± 2.21 | 104.49 ± 17.42 | 79.61 ± 9.20 |

| 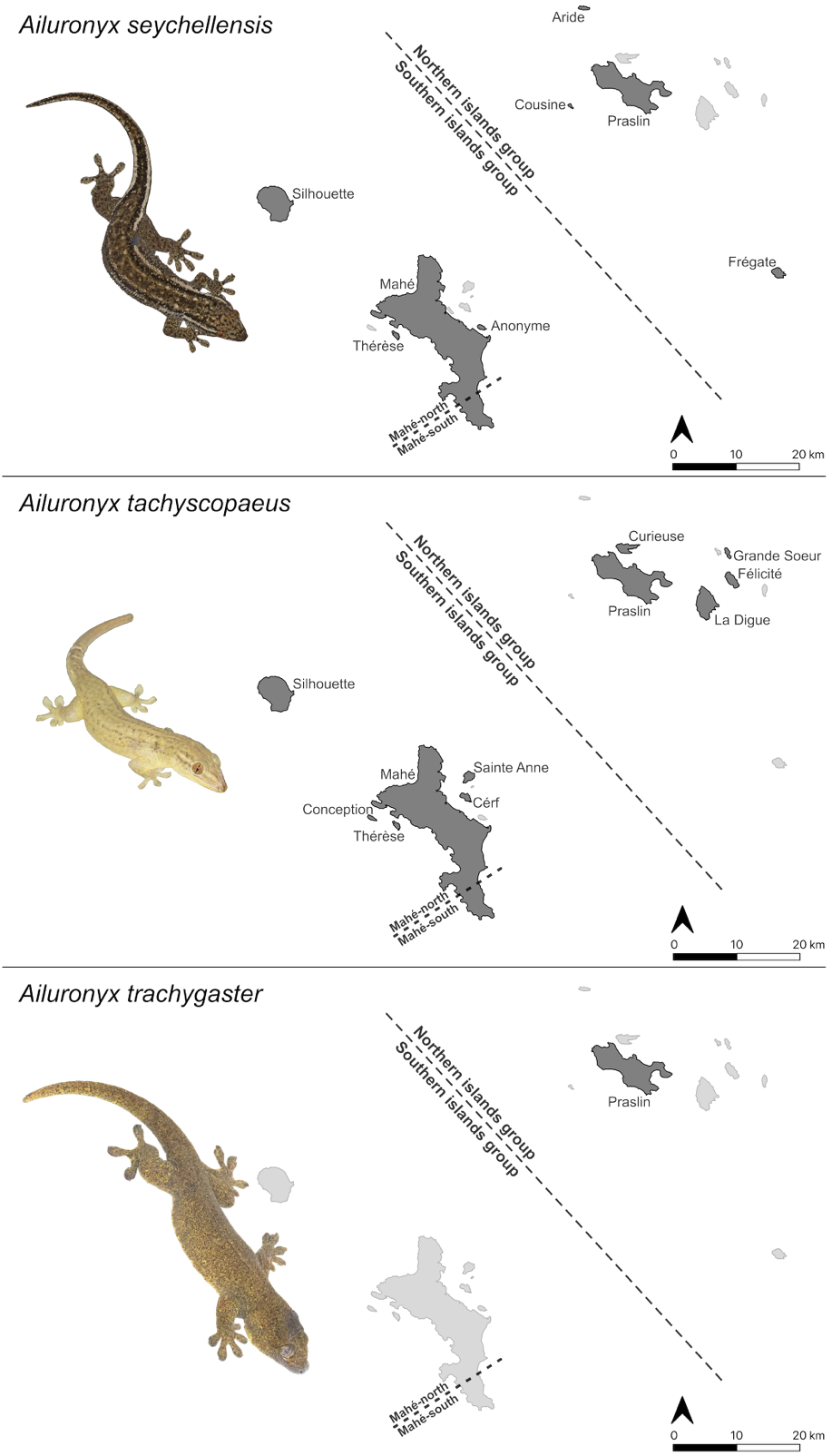 |
| --- |
| **Figure S1.** Distribution of each *Ailuronyx* species across the Seychelles granitic islands based on fieldwork conducted between January 2024 and January 2025. Dark grey islands (labelled) represent confirmed presence, while light grey islands indicate surveyed locations where the species was not detected. Islands not visited during this study are not shown; see Figure 1 for the complete set of surveyed islands and Table S1 for sampling effort per island. |

|  |
| --- |
| **Figure S2.** Comparison of the simplified surfaces derived from the two different segmentation approaches (Otsu’s local threshold, optimal global threshold). Colour gradient shows Hausdorff Distances. Voxel size of μCT volume is approximately 25 μm. Differences between the two thresholding methods are within a margin of 1-2 voxels. Specimen used for visual representation is *Ailuronyx trachygaster*, ZMFK-HERP-104233. |

|  |
| --- |
| **Figure S3.** Location of 120 and 48 fixed landmarks placed on the cranium (top) and mandible (bottom), respectively. A description of the landmarks is given in Table S2. Specimen used for visual representation is *Ailuronyx trachygaster*, ZMFK-HERP-104233. |

|  |
| --- |
| **Figure S4.** Principal Component Analysis to assess landmarking bias, with one individual per species being landmarked three times, for **A** – cranium and **B** – mandible, respectively. PCAs were generated separately for each species. |

|  |
| --- |
| **Figure S5.** Visualizations of minimum negative (min) and maximum positive (max) principal component scores using thin plate splines to illustrate shape deviations from the mean shape. X, Y shows the dorsal view, Y, Z shows the lateral view. Cranium: **A** – PC1 minimum, **B** – PC1 maximum, **C** – PC2 minimum, **D** – PC2 maximum. Mandible: **E** – PC1 minimum, **F** – PC1 maximum, **G** – PC2 minimum, **H** – PC2 maximum. |

| 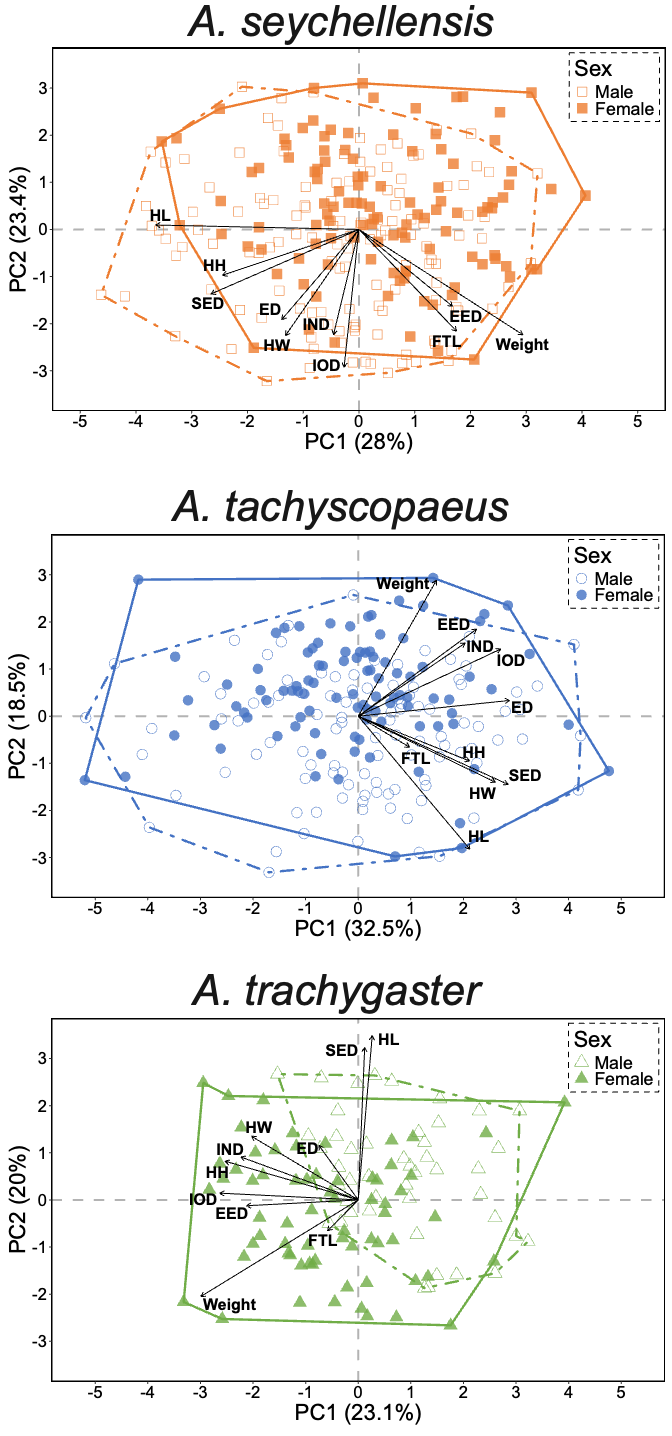 |
| --- |
| **Figure S6.** Principal Component Analysis of body-size corrected multivariate morphometric traits between the sexes of *A. seychellensis*, *A. tachyscopaeus* and *A. trachygaster*, respectively. Morphometric trait abbreviations used in the figure panel are eye diameter (ED), eye-ear distance (EED), fourth toe length (FTL), head height (HH), head length (HL), head width (HW), internarial distance (IND), interorbital distance (IOD) and snout-eye distance (SED). |
